# Bangla validation of the smartphone addiction scale and the academic underachievement scale: The associative role of classroom mindful attention in the relationship between smartphone addiction and underachievement among adolescents and young adults

**DOI:** 10.1371/journal.pone.0353865

**Published:** 2026-07-16

**Authors:** Shakira Khatun, Md. Mehedi Hassan Ridoy, Abu Hanif Noman, Md. Muazzem Hosen Sharon, Md. Rahim Mia, Jahedul Islam, Sajedul Hasan Himel, Mohd. Ashik Shahrier

**Affiliations:** 1 Department of Psychology, University of Rajshahi, Rajshahi, Bangladesh; 2 Department of Social Work, University of Rajshahi, Rajshahi, Bangladesh; University of Ghana, GHANA

## Abstract

This study aimed to validate the Smartphone Addiction Scale–Short Version (SAS-SV) and the Perceived Academic Underachievement Scale (PAUS) for Bangladeshi adolescents and young adults. In addition, the study investigated whether classroom mindful attention has an indirect association with the relationship between smartphone addiction and academic underachievement in the target population. A cross-sectional survey using convenience sampling was conducted with 712 participants recruited from different educational institutions in Bangladesh. Bangla versions of SAS-SV, Classroom Mindful Attention Regulation Scale, and PAUS were administered in collecting data. Factor structure, reliability, and validity of the measures were assessed, and statistical mediation to determine the indirect association was conducted to achieve the study aims. Both SAS-SV and PAUS showed excellent internal consistency (Cronbach’s *α* = 0.95 and 0.91, respectively) and confirmed the predefined factor structures with good model fits (CFI = 0.99, TLI = 0.99, RMSEA = 0.05, SRMR = 0.03; PAUS: CFI = 0.99, TLI = 0.99, RMSEA = 0.04, SRMR = 0.03). While determining the convergent validity of the measures, both smartphone addiction and perceived academic underachievement were associated with lower GPAs and reduced classroom mindful attention. Moreover, the SAS-SV and PAUS were found to be invariably applicable across age, gender, and data collection mode. Mediation analyses indicate that the self-awareness component of classroom mindful attention had an indirect association with the relationship between smartphone addiction and perceived academic underachievement in young adults (*B* = 0.014, 95% CI [0.001, 0.028], *p* = .033) and the combined sample (*B* = 0.020, 95% CI [0.005, 0.036], *p* = .009), whereas no significant indirect association was observed among adolescents. These findings highlight the associative role of classroom self-awareness in the relationship between smartphone addiction and perceived academic underachievement and underscore the potential role of mindfulness-based interventions in educational settings.

## Introduction

Over the past decade, smartphone ownership has risen dramatically, with approximately half of the global population now using these devices [1,2]. In high-income regions such as North America and Europe, ownership exceeds 80%, while usage in low- and middle-income countries continues to expand rapidly [3]. In this digital age, smartphones have become integral to daily life, shaping how people communicate, access information, and interact with the world around them [4]. By the end of 2022, over 6.6 billion people worldwide were using smartphones, and the number of users is expected to continue rising steadily over the coming years, approaching 7.8 billion by 2028 [5]. This global trend is also reflected in Bangladesh where smartphone access has expanded significantly due to improved internet availability and affordability [6]. Despite the growing availability of digital technologies, smartphones remain insufficiently integrated into structured educational practices in Bangladesh, a gap attributed to infrastructural limitations, lack of teacher training, and concerns about excessive screen exposure, particularly in rural areas [7]. This pattern is consistent with the evidence in developing countries, where, despite widespread smartphone access, their pedagogical use in higher education remains limited and underdeveloped [8]. In contrast, the global literature suggests that smartphone-based-e-learning has been systematically integrated into the educational systems through blended learning models, mobile applications, and interactive digital platforms, thereby enhancing accessibility and engagement in the teaching-learning process [9]. This highlights a clear gap between access to technology-based education and its effective implementation across cultures. In Bangladesh context, more than 90% of adolescents use smartphones, reflecting their widespread adoption and increasing reliance on digital technologies [6,10].

Smartphone usage among Bangladeshi students is restricted to entertainment and communication activities such as social media, gaming, and messaging rather than educational purposes [11]. A large percentage of young adult students, spending three to six hours daily on smartphones, has been found to be scolded for smartphone use [11], indicating teacher and parental concerns about inappropriate or excessive usage. Consistent with this, global literature suggests that parents could not control the smartphone overuse by their adolescent children despite the awareness of the detrimental impacts of smartphone overuse [12]. The majority of young adult students in higher educational institutions of Bangladesh use smartphones on an average of 6.5 hours per day for entertainment purposes only [13]. Similar patterns have also been observed among younger populations, where a substantial proportion of early adolescents spend at least three hours per day using smartphones or other digital devices [14]. Although smartphones offer numerous advantages, including communication, access to information, education, and entertainment [15,16], their excessive use has increasingly been linked to poor academic performance in urban Bangladeshi students due to the prioritization of social networking over academic applications [17,18].

Smartphone overuse leading to smartphone addiction has not been formally classified as a distinct clinical disorder by the World Health Organization; instead, it is often merged within a broader perspective of behavioral addictions, such as gaming disorder. But smartphone addiction is an emerging public health concern, indicating that up to 37% of adolescents show signs of it and exhibit maladaptive behavioral patterns characterized by withdrawal symptoms and interference with daily activities [19,20]. Excessive smartphone use has been linked to a range of psychological and behavioral difficulties, including depression, stress, fatigue, headaches, poor concentration, social isolation, and reduced sleep quality [21–26]. It may also reduce academic performance and engagement in learning [27–31]. In educational settings, smartphone use disrupts the learning process, as students engage in social media or other online communications during classes [32–34], leading to decreased attention, lower productivity, and impaired learning efficiency [35,36]. Higher levels of smartphone usage have also been associated with decreased concentration and ineffective time management [37]. Thus, despite its access to educational resources and effective communication, smartphone overuse frequently results in distraction, procrastination, and reduced academic engagement [38,39].

Parental guidance plays a crucial role in shaping adolescents’ smartphone use, with effective monitoring and balanced control helping to prevent excessive or addictive behaviors [40]. Specifically, authoritative parenting—characterized by high warmth combined with firm control—is associated with lower risks of smartphone addiction among adolescents [41]. Alongside parental influence, students’ self-control is equally important. Smartphone addiction is strongly linked to poor self-regulation, as individuals struggle to limit their usage despite its negative consequences [42]. Empirical evidence suggests that individuals with lower self-control—the inability to resist impulses and complete necessary tasks—are more likely to respond immediately to mobile notifications and have difficulty resisting the urge to check their smartphones [43,44]. Low self-control has also been linked to problematic smartphone behaviors, including late-night usage and sleep disruptions [45,46], as well as increased procrastination on social media platforms such as Facebook [47]. Prolonged social media engagement has been found to intensify smartphone dependency, further undermining academic performance [48–51]. Research suggests that such impulsive engagements are linked to deficits in attentional control, emotional regulation, and cognitive functioning in classroom settings [51,52].

In classroom settings, mindful attention has been shown to counteract distractions and enhance students’ ability to regulate attention, maintain focus, and engage fully with learning activities [53,54]. Mindfulness practices foster present-moment awareness, sensory engagement, and empathetic interactions in classrooms, supporting creativity, motivation, adaptability, and attentional control, ultimately promoting sustained engagement [55–59]. Students who do not engage mindfully often become inattentive to teacher instructions, discontinue academic tasks prematurely, lose focus during lessons, struggle to follow classroom norms, and display behaviors that disrupt learning [53,60,61]. Classroom mindfulness enhances awareness of thoughts, emotions, and bodily sensations, which can help reduce addictive behaviors and manage cravings [62]. Research also indicates that mindfulness practices can significantly reduce anxiety [35], thereby improving attention regulation, information retrieval, and the ability to perform goal-directed tasks through self-regulatory skills [63–65]. Taken together, practicing mindfulness is particularly important for promoting self-awareness, acceptance, self-regulation, and coping with academic stress [66,67]. A growing body of research has consistently linked mindfulness to lower levels of compulsive smartphone use [68,69]. By enhancing self-awareness and emotional regulation, mindfulness-based approaches have been shown to reduce maladaptive patterns of technology use [70]. From a theoretical perspective, self-regulation theory helps explain this relationship, as mindfulness supports attentional control, emotional regulation, and reflective awareness—components essential for strengthening self-regulatory mechanisms [71]. Individuals with higher mindfulness tend to demonstrate greater self-control and a more active regulatory system, reducing impulsive technology-related behaviors [72]. In addition, mindfulness has been found to be positively associated with self-control and negatively associated with rumination, both of which contribute to lower levels of problematic smartphone use among college students [69]. In addition, mindfulness has been shown to partially mediate the relationship between emotion regulation and mobile phone addiction, suggesting an important pathway linking mindfulness to technology use behaviors [73]. Previous studies have highlighted the need for more in-depth research on the role of mindfulness, especially in relation to academic stress and self-regulation [66]. Research has also documented a negative relationship between smartphone addiction and academic performance [51,74,75], with smartphone multitasking contributing to lower academic achievement [51]. However, the potential mediating role of classroom mindful attention in this relationship remains underexplored. Therefore, the present study aims to examine whether mindful attention in the classroom has an associative role in the relationship between smartphone addiction and academic underachievement among adolescents and young adults.

To examine this relationship, two standardized scales have been validated for use in Bangladesh. The Smartphone Addiction Scale (SAS; [76]) and its short version (SAS-SV; [76]) are among the most widely used instruments for assessing problematic smartphone use. Developed in collaboration with clinicians, these scales assess multiple aspects of behavioral addiction, including withdrawal, tolerance, loss of control, and continued use despite harmful consequences [77]. The full version demonstrates strong shared variance with related constructs, while the short form reliably predicts clinical judgments of smartphone addiction [76]. The 10-item SAS-SV, derived from the original 33-item version, has been translated and validated across multiple cultural contexts, such as Spain and France [78], Switzerland [16], Morocco [79], Turkey [80], Egypt [81], Indonesia [82], and Italy [83].The Perceived Academic Underachievement Scale (PAUS) was developed by Snyder and Adelson [84] in the USA to assess perceived academic underachievement among adults and was later adapted for use in Turkey by Bozgün et al. [85]. To ensure cultural appropriateness for Bangladeshi adolescents and young adults, this study validated the Bangla versions of the Smartphone Addiction Scale–Short Version (SAS-SV; [76]) and the Perceived Academic Underachievement Scale (PAUS; [84]). Therefore, the specific aims of the present study were: (1) to evaluate the factor structures and reliabilities of the Bangla versions of the Smartphone Addiction Scale–Short Form (SAS-SV) and the Perceived Academic Underachievement Scale (PAUS); (2) to assess the convergent validity of the scales by determining their relationships with relevant criterion measures; (3) To test whether the validated Bangla versions of the scales can be invariably be applied across age, gender, and data collection mode; and (4) to investigate whether mindful attention in the classroom has an indirect association with the relationship between smartphone addiction and academic underachievement among adolescents, young adults, or the combined sample.

## Materials and methods

### Participants

A total of 712 adolescents and young adults were recruited through convenience sampling method from various universities, colleges, and schools of Bangladesh. Educational institutions were selected based on accessibility, participants’ willingness to respond, and the administrative approval. Participants were approached for data collection through classroom visits and announcements, and those aged 19–27 years were invited to participate voluntarily. For participants under 18 years of age, parental and/or teacher consent was obtained prior to data collection. Participants’ ages ranged from 12 to 27 years, with a mean age of 21.31 ± 2.81 years (Mean ± SD). Among them, 345 (48.46%) were female and 367 (51.54%) were male. Because convenience sampling was used, the findings may have limited generalizability. However, data were collected from different institutions to enhance sample diversity. Data normality was checked with the predefined skewness and kurtosis cutoffs reported in the Data Analysis section, rather than inferred from sampling heterogeneity. Based on age classification, 179 (15.14%) participants were adolescents (12–18 years), while 533 (84.86%) were young adults (19–27 years). In terms of educational level, the majority were undergraduate students (n = 460, 64.61%), followed by higher secondary students (n = 139, 19.52%), secondary students (n = 76, 10.67%), and postgraduate students (n = 37, 5.20%). Regarding academic discipline, 501 (70.37%) participants were enrolled in non-science faculties (e.g., social sciences, humanities, and business studies faculties), while 210 (29.49%) were enrolled in science faculties (e.g., natural sciences and engineering faculties), representing distinct academic fields. Most participants were from rural areas (n = 530, 74.44%), with the remaining (n = 182, 25.56%) from urban areas. The majority of the participants were unmarried (n = 664, 93.26%). With respect to self-perceived socioeconomic status, the majority was identified as middle class (66.0%), followed by lower-middle class (21.06%), upper-middle class (4.77%), lower class (6.60%), and upper class (1.54%). Based on their grade point averages (GPAs) from the most recent exam, participants were categorized as high, medium, and low achievers. This classification followed the grading system of the University Grants Commission (UGC) of Bangladesh for young adults, and that of the Boards of Intermediate and Secondary Education, Bangladesh for adolescents. Participants with scores of 70% or above were classified as high achievers, those with scores from 60% to less than 70% as medium achievers, and those with scores from 40% to less than 60% as low achievers.

### Procedure

The recruitment of the participants was conducted between April 28 and June 30 2025. Data were collected using both offline and online methods. Offline data were obtained face-to-face using the questionnaires, with written informed consent obtained from each participant. For adolescents aged 12–18 years, parental consent was obtained whenever feasible. During school-based data collection, when parents were not available, permission to reach participants in the school setting was obtained from the institutional authority, in accordance with the procedures approved by the Ethical Review Committee–Research and Publication (ERCRP). In addition, all adolescent participants provided their own assent before participation. The online survey was administered digitally via Google Forms. A survey link was distributed to participants through online platforms, including Facebook, WhatsApp, and email. Participants completed an electronic informed consent form before beginning the survey, and only those who provided consent were allowed to participate. The survey took approximately 20–30 minutes to complete for each participant, and they were thanked afterward for their participation. Ethical approval for the study was granted by the Ethical Review Committee–Research and Publication (ERCRP), Department of Psychology, University of Rajshahi, Bangladesh [approval code: ERCRP-PSYRU-7(3)25]. The study also adhered to the ethical principles of the Declaration of Helsinki and its later amendments [86].

Prior to data collection, the original English versions of the Smartphone Addiction Scale–Short Version (SAS-SV) and the Perceived Academic Underachievement Scale (PAUS) were translated into Bangla following the International Test Commission (ITC) guidelines for translating and adapting tests [87]. Initially, four bilingual experts, proficient in both English and Bangla as well as experienced in psychological test development and adaptation, independently translated the scales. Their translations were reviewed, compared, and consolidated into a single Bangla version that preserved the original meaning and cultural relevance. To ensure semantic and conceptual equivalence, three additional bilingual experts back-translated the Bangla version of the scales into English. The back-translations were reviewed, edited, and carefully compared with the original English version of the scales. Necessary revisions were made to ensure that the scales accurately reflect both the concepts and the meaning of the original instruments. A pilot study was then conducted on 30 students (16 adolescents and 14 young adults) representing the same population of the main study (M = 22.43, SD = 1.63). Students assessed the linguistic clarity, cultural relevance, and contextual suitability of the translated scales. In the pilot sample, the presence of demographic characteristics such as age, gender, academic discipline, and others were the same as those of the main study. Most participants reported that the key concepts were clearly expressed, and the items were generally easy to understand and accurately captured the intended constructs. Based on their feedback, minor modifications in the measures were made to enhance clarity and cultural fit. Following the pilot study, the final instruments used for the main study included the SAS-SV (10 items, 6-point Likert scale), the PAUS (6 items, 5-point scale), and the Classroom Mindful Attention Regulation Scale (CMARS, 10 items), administered to the total sample (n = 712).

### Measures

**Smartphone Addiction Scale-Short Version (SAS-S*V*).** The Smartphone Addiction Scale–Short Version (SAS-SV; [76]) is a unidimensional self-report instrument designed to assess the risk of smartphone addiction and identify high-risk individuals. It conceptualizes smartphone addiction as excessive use that interferes with daily responsibilities and shows addiction-like symptoms such as tolerance, withdrawal, loss of control, cravings, and emotional instability [48,49]. The items of the SAS-SV were derived from the original 33-item Smartphone Addiction Scale (SAS; [48]). The SAS-SV consists of 10 items, each rated on a six-point Likert scale: *1 = strongly disagree, 2 = disagree, 3 = slightly disagree, 4 = slightly agree, 5 = agree, and 6 = strongly agree*. Total scores on the scale can range between 10 and 60. Higher scores indicate a greater risk of smartphone addiction. During its initial development, the scale showed excellent internal reliability, with a Cronbach’s alpha of 0.97. In the present study, the SAS-SV demonstrated excellent internal consistency reliabilities, with Cronbach’s alpha and McDonald’s omega values of 0.92 and 0.91 for the combined sample and 0.95 and 0.94 for the adolescent and young adult samples, respectively.

**Perceived Academic Underachievement Scale (PAUS).** The Perceived Academic Underachievement Scale (PAUS; [84]) assesses perceived academic underachievement among individuals in a particular course or overall coursework. Although originally developed for adults aged 18 and above, the scale was adapted for use with adolescents aged 12 and older in this study. It captures situations in which individuals feel that their performance does not reflect their true abilities, even though they know they could perform better [84]. The scale consists of 6 items rated on a 5-point Likert scale (*1 = strongly disagree, 2 = disagree, 3 = neutral, 4 = agree, 5 = strongly agree*), resulting in total scores ranging from 6 to 30. Lower scores reflect minimal or no perception of academic underachievement, whereas higher scores indicate a stronger perception of academic difficulties. In the PAUS, item 2 is reverse-coded. Prior to the main study, a pilot study conducted with 16 adolescents indicated that the items were clear, understandable, and appropriate for the target age group, with minor adjustments made to improve clarity without altering the original meaning. In the original study, the scale demonstrated excellent internal consistency, with a Cronbach’s alpha of 0.91. For the present sample, the scale also exhibited good internal consistency reliabilities with Cronbach’s alpha and McDonald’s omega values of 0.91 for the combined sample and 0.93 and 0.91 for the adolescent and young adult samples, respectively.

**Classroom Mindful Attention Regulation Scale (CMARS).** The Classroom Mindful Attention Regulation Scale (CMARS; [67]), consists of 10 items designed to assess emerging adults’ ability to nonjudgmentally regulate attention to present-moment stimuli in classroom settings. The scale captures two dimensions of mindful attention regulation: self-awareness and emotional regulation. Responses are recorded on a 5-point Likert scale, ranging from *1 (almost never) to 5 (almost always)*. Negatively worded items (2, 4, 5, 8, and 10) are reverse-scored to maintain consistency with the scale’s positive orientation. Higher scores indicate greater mindful attention regulation, while lower scores reflect weaker attentional control. In its original development, the CMARS demonstrated strong internal consistency, with *ω* = 0.83 for the total scale and *ω* = 0.82 and 0.75 for the emotional regulation and self-awareness subscales, respectively. In the present study, the total scale showed excellent reliability, with alpha and omega values of 0.85 for the total sample and 0.93 and 0.82 for the adolescent and young adult samples, respectively.

### Data analysis

The psychometric evaluation of the SAS-SV and PAUS incorporated both Classical Test Theory (CTT) and Item Response Theory (IRT) frameworks to ensure comprehensive scale validation. The validation process involved several components, including reliability assessment (internal consistency), construct validation through EFA and CFA, evaluation of convergent validity, and item-level examination using IRT models. All instruments used in this study—the SAS-SV, PAUS, and CMARS—recorded self-reported responses from adolescents and young adults, reflecting participants’ subjective assessment of smartphone overuse, perceived academic underachievement, and mindful attention in the classroom. To assess the data normality, the values of skewness (< 2) and kurtosis (< 7) were considered acceptable indicators [88]. Internal consistency was evaluated using Cronbach’s alpha (α) and McDonald’s omega (ω), with values ≥ 0.70 considered acceptable [89]. The mean inter-item correlations (ranging from 0.15 to 0.50 [90]) and corrected item-total correlations (≥ 0.30 [91]) indicated satisfactory item discrimination. Composite reliability (≥ 0.70) and average variance extraction (≥ 0.50) were obtained from CFA factor loadings [92].

Prior to exploratory factor analysis (EFA), sampling adequacy and data suitability were assessed. For the SAS-SV and PAUS, the Kaiser-Meyer-Olkin (KMO) measure (> 0.60 [93]), Bartlett’s test of sphericity value (*p* < .001 [93]), and determinant value (>.00001 [94]) satisfied the prerequisites for factor analysis. EFA with geominQ rotation was conducted for both scales, retaining factors with eigenvalues greater than one [95]. A parallel analysis was also performed to confirm the number of retained factors [96,97]. In confirmatory factor analysis (CFA), model fit was evaluated using multiple indices: χ²/df < 5 [98], Comparative Fit Index (CFI), Tucker-Lewis Index (TLI ≥ 0.95 [99]), Root Mean Square Error of Approximation (RMSEA), and Standardized Root Mean Square Residual (SRMR, ≤ 0.08 [100]). Multi-group CFA was run to check measurement invariance across age, gender, and data collection mode with ΔCFI ≤ 0.01 and ΔRMSEA ≤ 0.015 [101].

After establishing the optimal factor structure using confirmatory factor analysis (CFA), item response theory (IRT) analyses were performed using the graded response model (GRM; [102]) to evaluate item discrimination and threshold parameters after confirming IRT assumptions through local independence, monotonicity, and scalability of the SAS-SV and PAUS. Item discrimination and threshold parameters were estimated to assess how well each item differentiated between respondents at different levels of the latent traits. According to Baker and Kim [103], discrimination values of 0.10–0.34 indicate very low, 0.35–0.64 low, 0.65–1.34 moderate, 1.35–1.69 high, and values above 1.70 indicate very high discrimination. Threshold parameters represent the level of the latent trait (θ) required to move from one response category to the next [104]. To ensure that the items functioned independently, local independence was evaluated using residual correlation coefficients (Q3 statistics; [105]), with values below 0.20 indicating no local dependence [106]. Item scalability was assessed using Loevinger’s H coefficients [107], where values ≥ 0.50 indicate strong scalability, 0.40–0.49 moderate, and 0.30–0.39 weak [108]. Monotonicity was examined via the monotonicity index, with values below 40 considered acceptable [109]. The GRM was performed using R package *ltm* version 1.2.0. The scalability and monotonicity were determined using the R package *mokken* version 3.1.2. Local independence was checked through the R package *mirt* version 1.44.0. In addition, item information curves (IICs) and the test information function (TIF) were generated to assess the precision of the scales across different levels of the latent traits. Convergent validity of the SAS-SV and PAUS was assessed by examining their associations with the Bangla version of the Classroom Mindful Attention Regulation Scale (CMARS) and the obtained GPAs of the participants. Furthermore, to have a more nuanced interpretation of results, we have examined whether smartphone addiction, perceived academic underachievement, and mindful attention in the classroom differ by age (12–18/19–27), gender (male/female), academic field (science/non-science), and academic achievement (high/medium/low achievers). Statistical mediation analysis was used to assess whether mindful attention in the classroom has an indirect association with the relationship between smartphone addiction and perceived academic underachievement. Data were processed and analyzed using the IBM SPSS (version 26), RStudio (version 2025.09.1 + 401), JASP (version 0.95.2, Intel) and Microsoft Excel 365.

## Results

### Descriptive statistics and item-level properties of the Bangla versions of SAS-SV and PAUS

Descriptive statistics presented in Table 1 showed that the mean scores for individual items on the SAS-SV ranged from 3.35 (SD = 1.41) to 3.80 (SD = 1.53), whereas for the PAUS, item means varied between 3.22 (SD = 1.04) and 3.48 (SD = 1.19; 1.24). Analyses of item-level distribution characteristics revealed that skewness values were within acceptable limits, ranging from −0.19 to 0.05 for SAS-SV and from −0.48 to −0.15 for PAUS. Similarly, kurtosis values fall between −1.09 and −0.69 for SAS-SV and between −0.85 and −0.52 for PAUS, all within the recommended thresholds of less than 2 for skewness and less than 7 for kurtosis [60]. Corrected item-total correlations demonstrated good item discrimination, ranging between 0.73 (Item 8) and 0.80 (Item 6) for SAS-SV, and from 0.65 (Item 1) to 0.81 (Item 4) for PAUS (Table 1).

**Table 1:** Item-level psychometric properties of the Bangla SAS-SV and Bangla PAUS.

	Mean	SD	Skewness	Kurtosis	CITC
Bangla Smartphone Addiction Scale-Short Version (SAS-SV)					
Item 1	3.61	1.48	0.02	−1.02	0.76
Item 2	3.80	1.53	−0.19	−1.09	0.80
Item 3	3.60	1.49	−0.06	−0.93	0.74
Item 4	3.47	1.40	−0.12	−0.78	0.77
Item 5	3.51	1.41	−0.16	−0.78	0.79
Item 6	3.45	1.45	−0.12	−0.90	0.80
Item 7	3.35	1.41	−0.04	−0.88	0.78
Item 8	3.36	1.42	0.05	−0.69	0.73
Item 9	3.63	1.48	−0.10	−0.85	0.79
Item 10	3.72	1.51	−0.06	−0.92	0.77
Bangla Perceived Academic Underachievement Scale (PAUS)					
Item 1	3.24	1.09	−0.15	−0.57	0.65
Item 2	3.36	1.14	−0.30	−0.85	0.77
Item 3	3.22	1.04	−0.29	−0.52	0.67
Item 4	3.40	1.16	−0.39	−0.82	0.81
Item 5	3.48	1.19	−0.44	−0.79	0.80
Item 6	3.48	1.24	−0.48	−0.79	0.79

Note. M = Mean, SD = Standard Deviation, CITC = Corrected Item-Total Correlation.

### Reliability evidence

The internal consistency reliabilities of both scales were excellent (Table 2), as reflected by Cronbach’s alphas (0.95 for the Bangla SAS-SV and 0.91 for the Bangla PAUS) and McDonald’s omegas (0.95 for SAS-SV and 0.91 for PAUS). The mean item-total correlations were 0.64 for SAS-SV and 0.62 for PAUS. Composite reliability estimates were also high (0.96 for SAS-SV and 0.91 for PAUS; Table 2).

**Table 2:** Scale-level psychometric properties of the Bangla SAS-SV and Bangla PAUS.

Psychometric properties	SAS-SV	PAUS	Suggested cut offs
Mean inter-item correlation	0.64	0.62	Between 0.15 and 0.50
McDonald’s Omega	0.95	0.91	≥ 0.70
Cronbach’s Alpha	0.95	0.91	≥ 0.70
Composite Reliability	0.96	0.91	≥ 0.70
Average Variance Extracted (AVE)	0.66	0.66	≥ 0.50

### Dimensionality

An exploratory factor analysis (EFA) was conducted to investigate the underlying factor structure of the Bangla versions of SAS-SV and PAUS. Prior to this, the Kaiser-Meyer-Olkin (KMO) measures of sampling adequacy were tested and found excellent (0.96 for SAS-SV; 0.91 for PAUS). Bartlett’s tests of sphericity values were also significant (*χ²* = 5298.62, *p* < .001 for SAS-SV; *χ²* = 2638.98, *p* < .001 for PAUS), indicating that the data were appropriate for factor analysis. The EFA results revealed that both instruments demonstrated a unidimensional factor structure, accounting for 63.8% and 62.9% of the variances for SAS-SV and PAUS, respectively. The analyses employed the minimal residual method with oblique (geominQ) rotation. To confirm the number of factors retained, parallel analysis was conducted, which demonstrated that the observed eigenvalues from the real data exceeded those from simulated datasets, confirming a single-factor solution for both measures [68,69].

### Fit indices of measurement models

To validate the factor structure of the SAS-SV and PAUS, confirmatory factor analysis (CFA) was performed using the diagonally weighted least square (DWLS) estimation method (Table 3). The fit indices indicated excellent model fits according to established criteria. Specifically, the single-factor models demonstrated a good fit to the data, with values as follows: for SAS-SV, *χ²/df* = 2.63, CFI = 0.99, TLI = 0.99, RMSEA = 0.05, and SRMR = 0.03; for PAUS, *χ²/df* = 2.26, CFI = 0.99, TLI = 0.99, RMSEA = 0.04, and SRMR = 0.03. Item factor loadings from the EFA for SAS-SV ranged from 0.75 to 0.83, while CFA loadings were slightly higher, ranging from 0.77 to 0.84. For PAUS, EFA factor loadings ranged from 0.69 to 0.86, whereas CFA factor loadings ranged between 0.71 and 0.87 (Table 4).

**Table 3:** Model fit indices of the Bangla versions of SAS-SV and PAUS using CFA.

Fit indices	SAS-SV	PAUS	Suggested cut offs
*χ* ^*2*^ */df*	2.63	2.26	< 5
Comparative fit index (CFI)	0.99	0.99	≥ 0.95
Tucker Lewis Index (TLI)	0.99	0.99	≥ 0.95
Root mean square error of approximation (RMSEA)	0.05	0.04	≤ 0.08
Standardized root mean square residual (SRMR)	0.03	0.03	≤ 0.08

Note*. χ²* = chi-square, *df* = degrees of freedom.

**Table 4:** Factor loadings of the items of Bangla SAS-SV and Bangla PAUS.

Scales	Items	Factor Loadings	
		EFA	CFA
SAS-SV	1. Missing planned work due to smartphone use.	0.79	0.80
	2. Having a hard time concentrating in class, while doing assignments, or while working due to smartphone use.	0.83	0.84
	3. Feeling pain in the wrists or at the back of the neck while using a smartphone.	0.77	0.78
	4. Won’t be able to stand not having a smartphone.	0.80	0.81
	5. Feeling impatient and fretful when I am not holding my smartphone.	0.81	0.83
	6. Having my smartphone in my mind even when I am not using it.	0.83	0.84
	7. I will never give up using my smartphone even when my daily life is already greatly affected by it.	0.81	0.82
	8. Constantly checking my smartphone so as not to miss conversations between other people on Twitter or Facebook.	0.75	0.77
	9. Using my smartphone longer than I had intended.	0.82	0.83
	10. The people around me tell me that I use my smartphone.	0.79	0.81
PAUS	1. I am performing below my capability in course.	0.69	0.71
	2. I am achieving to the maximum of my capability in course.	0.81	0.83
	3. To be honest, I feel that I am underachieving in course.	0.71	0.72
	4. I am performing below my ability in course.	0.86	0.87
	5. I could perform much better in course than I am currently performing.	0.85	0.87
	6. My achievement in course does not reflect how well I am capable of achieving in that class.	0.84	0.86

Note. CFA = Confirmatory Factor Analysis, EFA = Exploratory Factor Analysis.

### IRT assumptions and model evaluation

Following the confirmatory factor analysis, the assumptions of Item Response Theory (IRT) were evaluated to assess the unidimensional structure of the SAS-SV and PAUS. The residual correlation coefficients (Q3 statistics) ranged from −0.28 to 0.12 for SAS-SV and from −0.34 to 0.07 for PAUS, with none exceeding the commonly used threshold of 0.20. This indicates that the items met the assumption of local independence. Loevinger’s H coefficients (item scalability) ranged from 0.63 to 0.69 for the SAS-SV, with a scale-level H of 0.66 (SE = 0.01), and from 0.60 to 0.70 for the PAUS, with a scale-level H of 0.66 (SE = 0.01), suggesting strong scalability and acceptable item discrimination. The monotonicity analysis showed that all items in both scales demonstrated acceptable monotonicity, with minimal and no critical violations observed for most items. These findings collectively support the application of an IRT model for the SAS-SV and PAUS, confirming that the items perform well in measuring the underlying latent traits of smartphone addiction and perceived academic underachievement. In the graded response model (GRM), item discrimination parameters ranged from 2.01 to 2.65 for the SAS-SV (Mean *α* = 2.55), and from 1.70 to 3.12 for the PAUS (Mean *α* = 2.51; Table 5). All items on both measures showed very high discrimination power.

**Table 5:** Discrimination (*α*) and difficulty (b) parameters of Bangla SAS-SV and Bangla PAUS items using the IRT model.

	Bangla SAS-SV						Bangla PAUS					
	b_1_	b_2_	b_3_	b_4_	b_5_	*α*		b_1_	b_2_	b_3_	b_4_	*α*
Item 1	−2.25	−0.78	−0.06	0.85	1.42	2.19	Item 1	−2.25	−1.01	0.43	1.55	1.70
Item 2	−2.07	−0.99	−0.13	0.48	1.24	2.51	Item 2	−2.04	−0.81	0.07	1.19	2.55
Item 3	−2.00	−0.94	−0.06	0.84	1.51	2.01	Item 3	−2.29	−1.01	0.3	1.85	1.75
Item 4	−1.83	−0.94	0.03	0.94	1.73	2.35	Item 4	−1.89	−0.81	−0.03	1.11	3.11
Item 5	−1.84	−0.94	−0.05	0.91	1.70	2.43	Item 5	−1.87	−0.94	−0.05	−0.95	2.99
Item 6	−1.69	−0.84	0.10	0.84	1.66	2.65	Item 6	−1.75	−0.92	−0.09	−0.89	2.92
Item 7	−1.74	−0.73	0.15	0.95	1.77	2.48						
Item 8	−1.81	−0.85	0.14	1.19	1.75	2.01						
Item 9	−1.90	−0.99	−0.17	0.84	1.37	2.40						
Item 10	−2.07	−1.04	−0.18	0.79	1.23	2.23						

Note*. α* = Discrimination parameters. b_1_*-*b_4_/b_5_ = Difficulty parameters

The difficulty parameters in Table 5 reveal that the negative thresholds (b_1_–b_2_ for both scales) represent easier endorsement, whereas the positive thresholds (b_3_–b_5_ for SAS-SV; b_3_–b_4_ for PAUS) indicate progressively more difficult endorsement. Among the SAS-SV items, Item 1 had the lowest thresholds and was the easiest to endorse, while Item 7 had the highest thresholds, reflecting higher levels of the trait. For the PAUS, Item 6 had the lowest thresholds, indicating easiest endorsement, whereas Item 3 showed the highest thresholds, reflecting greater trait levels (Table 5). Overall, the distribution of thresholds across items demonstrates that both scales effectively capture responses from individuals with lower and higher levels of the underlying constructs.

**Fig 1:**
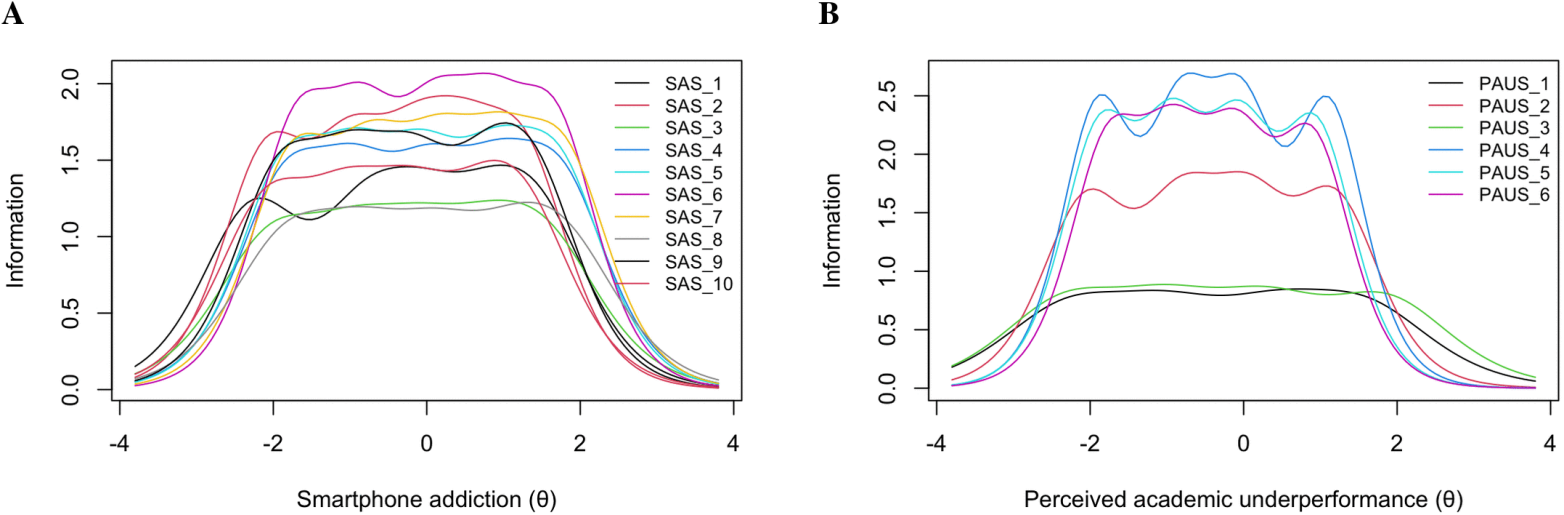
Item Information Curves (IICs) for the Bangla SAS-SV and Bangla PAUS as shown in panels A and B, respectively.

Fig 1 presents the item information curves (IICs), which illustrate how much information each item contributes across different levels of the underlying latent trait. In Panel A (SAS-SV), Item 6 emerged as the most informative, although other items also provided considerable information, particularly around the mid-range of the trait continuum. In Panel B (PAUS), Item 4 yielded the highest level of information, with the remaining items contributing meaningful information across a broad range of the latent trait.

**Fig 2:**
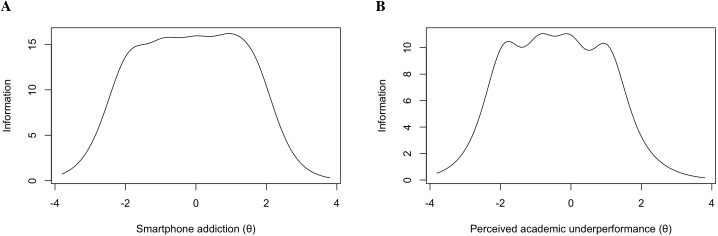
Test information functions of Bangla SAS-SV and Bangla PAUS as illustrated in Panel A and Panel B, respectively.

Fig 2 illustrates the test information functions (TIFs) for the Bangla versions of SAS-SV and PAUS. As shown in Panel A, the Bangla SAS-SV offers the highest level of information for θ values between −2.0 and 2.0. Similarly, Panel B shows that the Bangla PAUS also provides the most information within this θ range. Both scales demonstrate strong measurement precision at moderate levels of the latent trait, indicating their effectiveness in capturing variance around the average trait levels.

### Group-based invariance testing

A multi-group confirmatory factor analysis was conducted to examine the measurement invariance of the Bangla versions of SAS-SV and PAUS across age, gender, and data collection mode. The ΔCFI (≤ 0.010) and ΔRMSEA (≤ 0.015) values reported in Table 6 were within the recommended cutoffs, indicating that both scales are invariant and can be applied consistently across different age groups, between males and females, and irrespective of mode effects (online/offline data collection mode).

**Table 6:** Measurement invariance of the Bangla SAS-SV and Bangla PAUS across age, gender, and data collection mode.

		Model fits						Model comparison	
		*χ2*	*df*	*p*	CFI	TLI	RMSEA	ΔCFI	ΔRMSEA
Age (12-18/19-24)									
Configural	SAS-SV	109.33	70		0.999	0.999	0.040		
	PAUS	20.23	18		1.000	1.000	0.019		
Metric	SAS-SV	146.03	79	<.001	0.999	0.998	0.049	0	-0.009
	PAUS	83.16	13	<.001	0.996	0.995	0.086	0.004	-0.067
Scalar	SAS-SV	174.68	118	<.001	0.999	0.999	0.037	0	0.012
	PAUS	53.63	40	0.073	0.999	0.999	0.031	-0.003	0.055
Gender (Male/Female)									
Configural	SAS-SV	119.89	70		0.999	0.999	0.045		
	PAUS	25.67	18		0.999	0.999	0.035		
Metric	SAS-SV	155.96	79	<.001	0.998	0.998	0.052	0.001	-0.007
	PAUS	29.48	23	0.164	0.999	0.999	0.028	0	0.007
Scalar	SAS-SV	155.23	118	0.012	0.999	0.999	0.030	-0.001	0.022
	PAUS	64.99	40	<0.01	0.998	0.998	0.042	0.001	-0.014
Data Collection Mode (Online/Offline)									
Configural	SAS-SV	223.74	79		0.991	0.991	0.079		
	PAUS	45.37	18		0.997	0.995	0.065		
Metric	SAS-SV	188.03	79	< .001	0.995	0.994	0.062	-0.004	0.017
	PAUS	49.81	23	< .001	0.997	0.997	0.057	0	0.008
Scalar	SAS-SV	239.61	118	< .001	0.995	0.996	0.054	-0.001	0.008
	PAUS	93.14	40	< .001	0.995	0.996	0.061	0.002	-0.004

**Note:** *χ²* = Chi-square; *df* = degrees of freedom; *p* = significance value; CFI = Comparative Fit Index; TLI = Tucker–Lewis Index; RMSEA = Root Mean Square Error of Approximation; ΔCFI = change in Comparative Fit Index; ΔRMSEA = change in Root Mean Square Error of Approximation.

### Differences in SAS-SV, PAUS, and CMARS across academic achievement level, gender age, and academic field

A one-way ANOVA was conducted to examine differences in smartphone addiction (SAS-SV), classroom mindful attention regulation (CMARS), and perceived academic underachievement (PAUS) across levels of academic achievement (high/medium/low) of the participants. Results indicated no significant differences in SAS scores among high (M = 34.94, SD = 12.27), medium (M = 36.64, SD = 11.14), and low achievers (M = 36.35, SD = 11.87; *F* (2, 709) = 1.47, *p* = .231, η²p = .004]. Similarly, CMARS scores did not differ significantly across high (M = 27.34, SD = 8.63), medium (M = 26.06, SD = 6.97), and low achiever groups (M = 26.95, SD = 6.99; *F* (2, 709) = 1.52, *p* = .219, η²p = .004]. PAUS scores revealed no significant differences across achievement levels (high: M = 19.91, SD = 5.94; medium: M = 20.64, SD = 5.04; low: M = 20.81, SD = 5.55; *F* (2, 709) = 1.56, *p* = .210, η²p = .004]. Post hoc comparisons using Bonferroni corrections revealed no significant pairwise differences.

Independent samples t-tests were conducted to examine differences by gender, age group, and academic field. Male and female students did not differ significantly in SAS [*t* (710) = −0.776, *p* = .438], CMARS [*t* (710) = −0.171, *p* = .864], and PAUS scores [*t* (710) = −0.328, *p* = .743]. In respect of age group, adolescents (12–18 years) and young adults (19–24 years) did not differ significantly in SAS [*t* (710) = −1.219, *p* = .223], CMARS [*t* (710) = 0.035, *p* = .972], and PAUS scores [*t* (710) = −0.334, *p* = .739]. While considering academic filed, students from science and non-science streams had no differences in SAS [*t* (710) = 0.791, *p* = .429], CMARS [*t* (710) = 1.272, *p* = .204], and PAUS scores [*t* (710) = 0.332, *p* = .740]. Overall, these results suggest that smartphone addiction, classroom mindful attention, and perceived academic underachievement remain relatively stable by demographic characteristics and academic achievement levels.

### Convergent validity evidence

While considering the convergent validity of Bangla versions of the SAS-SV and PAUS, CMARS (classroom mindful attention regulation) and GPAs were used as criterion variables to determine the relationships of SAS-SV and PAUS with these variables. The results showed strong negative associations of CMARS with SAS-SV (*r* = −0.62, *p* < .001) and PAUS (*r* = −0.60, *p* < .001), indicating that higher smartphone addiction and perceived academic underachievement are associated with lower classroom mindful attention. Additionally, both SAS-SV (*r* = −0.08, *p* < .05) and PAUS (*r* = −0.08, *p* < .05) showed weak but statistically significant negative correlations with GPAs, indicating that higher smartphone use and perception of academic underachievement are linked to slightly lower academic performance (GPAs). These findings support the convergent validity of SAS-SV and PAUS, as they are meaningfully related to classroom mindful attention and GPAs. In addition, the AVE values for both scales were 0.66, aligning with the recommended thresholds, and further indicate the convergent validity of the measures.

### Indirect association of classroom mindful attention with the relationship between smartphone addiction and perceived underachievement

Statistical mediation analysis was conducted to examine whether the two components of classroom mindful attention—self-awareness (CMARS-SA) and emotional regulation (CMARS-ER)—were indirectly associated with the relationship between smartphone addiction (SAS-SV) and perceived academic underachievement (PAUS) across adolescents, young adults, or the combined sample. Parameters were estimated using the full information maximum likelihood (FIML), which provides unbiased estimates under the assumption of missing at random (MAR; [110,111]). Indirect effects were evaluated using the bootstrap resampling with percentile confidence intervals [112].

Among adolescents, the indirect associations through CMARS-SA (*B* = 0.051, 95% CI [−0.017, 0.127], *p* = .082) and CMARS-ER (*B* = 0.040, 95% CI [−0.019, 0.111], *p* = .163) remained statistically non-significant, indicating that classroom mindful attention components were not significantly associated with the relationship between smartphone addiction and perceived academic underachievement. However, the direct association between smartphone addiction and perceived academic underachievement was significant (*B* = 0.337, 95% CI [0.257, 0.403], *p* < .001).

Among young adults, the indirect association through CMARS-SA was statistically significant (*B* = 0.014, 95% CI [0.001, 0.027], *p* = .033), indicating that smartphone addiction was linked with perceived academic underachievement via classroom self-awareness in this cross-sectional model. This finding should not be interpreted as evidence that smartphone addiction causes lower classroom self-awareness, or that reduced classroom self-awareness leads to perceived academic underachievement. However, the indirect association through CMARS-ER was not significant (*B* = −0.001, 95% CI [−0.011, 0.010], *p* = .948). The direct association between smartphone addiction and perceived academic underachievement remained significant (*B* = 0.401, 95% CI [0.380, 0.423], *p* < .001).

In the combined sample, the indirect association through CMARS-SA was significant (*B* = 0.020, 95% CI [0.005, 0.035], *p* = .009), whereas the indirect association through CMARS-ER was not significant (*B* = 0.005, 95% CI [−0.008, 0.019], *p* = .428). The direct association between smartphone addiction and perceived academic underachievement was quite strong and significant (*B* = 0.393, 95% CI [0.372, 0.414], *p* < .001). Overall, these findings indicate that self-awareness is indirectly associated with the relationship between smartphone addiction and perceived academic underachievement, particularly among young adults and in the combined sample, whereas emotional regulation was not significantly associated with this relationship across adolescents, young adults, or the combined sample.

## Discussion

This study aimed to explore whether classroom mindful attention has an indirect association with the relationship between smartphone addiction and academic underachievement of adolescents and young adults in Bangladesh. To address this, two standardized instruments were used, the Smartphone Addiction Scale–Short Version (SAS-SV) for assessing smartphone addiction and the Perceived Academic Underachievement Scale (PAUS) for measuring self-perceived academic underachievement. The findings demonstrated that the higher levels of smartphone addiction were found to be associated with greater perceived academic underachievement, while classroom mindful attention—particularly self-awareness—was indirectly associated with this relationship. Although the SAS-SV and PAUS have been validated in multiple cultural contexts—such as Italian, Turkish, Chinese, and Serbian populations for the SAS-SV [83,113–115], and American and Turkish populations for the PAUS [84,85]—they have not yet been validated in Bangladesh.

To this end, the present study examined the factor structure and validated the Smartphone Addiction Scale–Short Version (SAS-SV; [76]) and the Perceived Academic Underachievement Scale (PAUS; [84]) among adolescents and young adults in Bangladesh. In the current study, the instruments were first translated and back-translated, followed by a pilot study with a small group of students to ensure linguistic equivalence, item clarity, and appropriate item directionality. Subsequently, a larger sample of adolescent and young adult students, recruited through convenience sampling, participated in the main validation study. The results indicated excellent internal consistency for both scales, consistent with previous research demonstrating high reliability for the SAS-SV across different cultural contexts, including the Iranian version (Cronbach’s *α* = 0.85; [116]), the Spanish version (*α* = 0.88; [78]), the Mexican version (*α* = 0.89; [117]) and the Serbian version (*α* = 0.89; [113]). Similarly, the PAUS showed strong reliability in the Turkish version (Cronbach’s α = 0.80; McDonald’s *ω* = 0.80; [85]) and the American version (*α* = 0.91; [84]). The excellent reliability estimates in this study further suggest that the Bangla SAS-SV and PAUS captured their intended constructs with a high degree of homogeneity. However, very high reliability coefficients, particularly those approaching or exceeding 0.95, may suggest some overlap in item content. Although the present psychometric evidence, including the factor analytic and IRT findings, supports retaining all items, future studies may further examine the potential for item redundancy using complementary psychometric approaches and independent samples. Exploratory factor analysis (EFA) confirmed that the data for both the SAS-SV and PAUS were suitable for factor analysis, with all items loading strongly onto a single factor. Parallel analysis further supported a unidimensional structure for both scales. These results are consistent with prior research demonstrating robust unidimensional structures for the SAS-SV in Chinese [115], Turkish [80], and Italian versions [118], as well as for the PAUS in the American version [84]. Confirmatory factor analysis (CFA) further validated the factor structures, with fit indices indicating an excellent fit to the data. These findings are consistent with previous studies reporting good model fit for the SAS-SV in Turkish [80], Chinese [84], and Korean versions [76], and for the PAUS in the American version [84]. Finally, the IRT analyses conducted in this study further support the psychometric robustness of both the SAS-SV and the PAUS. For the SAS-SV, the items demonstrated high scalability and discrimination, and the test information function showed that the scale provided the most information across varying ability ranges of the latent trait, indicating optimal measurement precision within this range. This pattern aligns with the findings of Hidalgo-Fuentes et al. [119], who also observed that the SAS-SV maintains consistent information across the latent continuum, with the highest reliability observed around the mean to two standard deviations above it. Although no previous studies have applied IRT analysis to the PAUS, the current findings suggest that it performed effectively in capturing the latent construct at varying levels of perceived academic underachievement.

Furthermore, multi-group confirmatory factor analysis confirmed measurement invariance across gender, age, and data collection mode, indicating that the Bangla versions of SAS-SV and PAUS function invariably across these groups. This suggests that the instruments maintain consistent psychometric properties regardless of mode effects (online/offline data collection), thereby strengthening their applicability for cross-cultural validations across diverse populations. In addition, the study findings are consistent with prior research supporting the factorial stability of the SAS-SV across different cultural and demographic contexts. For instance, a Honduran validation study reported full invariance across sex and age categories [119], while a Peruvian adolescent sample similarly demonstrated invariance across age, sex, and region [120]. Likewise, Servidio et al. [118] identified configural and metric invariance—and partial scalar invariance—across gender and age for a 9-item version of the SAS-SV, which closely aligns with the present findings. With regard to the PAUS, although this study is among the first to examine its measurement invariance, the results indicated that it operates equivalently across gender and age groups. However, previous research suggests that gender differences may influence how students perceive their academic performance. For example, Vera Gil [121] found that gender moderates the relationship between psychological resilience and academic performance, with female students often perceiving their academic abilities differently than male students. Similarly, Wang and Yu [122] reported that gender moderates the relationship between academic self-concept and achievement, motivation, and self-efficacy, indicating that males and females may evaluate their academic competence in distinct ways. Behera and Seth [123] reported no significant gender differences in smartphone addiction among university students, although academic discipline was identified as a significant predictor. This suggests that smartphone use patterns and perceived academic abilities vary across cultural and demographic contexts. The present study findings further revealed that smartphone addiction, classroom mindful attention, and perceived academic underperformance did not differ significantly across academic achievement levels, gender, age groups, or academic streams of the participants, highlighting that these variables were relatively consistent across key student subgroups.

This study revealed a strong negative correlation of classroom mindful attention regulation with the SAS-SV and PAUS, indicating that higher levels of smartphone addiction and perceived academic underachievement are associated with lower classroom mindful attention. This finding aligns with meta-analytic and empirical evidence showing that problematic smartphone use is inversely related to mindfulness and attentional control [124–126]. Additionally, previous research has demonstrated that greater mindfulness is significantly associated with better academic achievement, highlighting the role of attentional regulation in academic performance [127]. Studies have also shown that smartphone addiction can lead to decreased mindful attention in classroom settings [128]. Engagement with social media for entertainment can increase cognitive distractions, further reducing mindful attention in classroom settings [15,129–131]. Although the associations of SAS-SV and PAUS with academic performance (i.e., GPAs) were statistically significant, the effect sizes were small, indicating a weaker association. This suggests that higher smartphone addiction and perceived academic underachievement are associated with lower GPAs, but the strength of these relationships is limited. In a broader sense, these findings are consistent with prior research linking higher smartphone addiction and perceived academic underachievement to lower GPAs and exam scores [84,131–138], although effect sizes reported in the literature vary across studies. Collectively, these results support the convergent validity of the SAS-SV and PAUS, suggesting that both scales are meaningfully associated with attentional regulation and academic performance.

Furthermore, smartphone addiction was found to be significantly associated with perceived academic underachievement, with classroom mindful attention showing an indirect association in this relationship. Specifically, smartphone addiction was significantly associated with perceived academic underachievement through the self-awareness component of classroom mindful attention among young adults and in the combined sample. Contrary to it, emotional regulation did not show a significant relationship within the same analytical framework. This finding aligns with prior research indicating that excessive smartphone use is associated with negative academic outcomes, such as increased academic burnout and decreased engagement [139,140]. Previous studies have also reported that higher levels of smartphone addiction are associated with poorer concentration and lower academic performance among students [141,142]. Similarly, research evidence suggests that higher levels of mindfulness are linked to lower problematic smartphone use, greater self-awareness, better emotional regulation, and more favorable academic outcomes [73,143]. In addition, higher self-awareness has been associated with better self-monitoring of smartphone use and more favorable academic outcomes [144,145]. These associations are consistent with self-regulation theory, which emphasizes the capacity to manage attention, prioritize long-term goals, and inhibit distractions [146,147]. Developmental differences in self-regulation have been reported across age groups, and evidence suggests that age may moderate the relationship between self-regulation and smartphone addiction, with older adolescents and young adults typically demonstrating stronger regulatory capacities [148]. Additionally, the association between self-regulation and academic achievement tends to strengthen with age, particularly among university students. This is consistent with research indicating that self-regulatory abilities develop alongside cognitive maturity [149,150].

Older adolescents and young adults have also been reported to exhibit stronger self-monitoring, greater perceived social support, and better behavioral regulation than younger individuals [149,150]. Although these characteristics may support more effective self-regulation, the absence of a significant indirect association of emotional regulation influencing smartphone addiction and perceived academic underachievement in this study suggests that managing emotions alone may be insufficient to offset the academic challenges linked to smartphone overuse, particularly when self-regulatory awareness is low. Overall, these findings suggest that self-awareness serves as a correlate linking smartphone addiction and perceived academic underachievement. However, given the cross-sectional nature of the study, this relationship should not be explained as a causal mechanism.

The findings of this study have practical implications for educational and psychological interventions aimed at mitigating the negative effects of smartphone addiction on academic performance. This highlights the importance of fostering self-awareness in students, which can be achieved through mindfulness-based activities such as guided attention exercises, reflective journaling, or classroom mindfulness practices. Although the indirect effects through classroom self-awareness were statistically significant, their relatively small magnitude (*B* = 0.014 in young adults and *B* = 0.020 in the combined sample) indicates that classroom self-awareness accounts for only a modest statistical association within the observed relationships. Therefore, classroom self-awareness should be considered a modest contributing factor statistically associated with perceived academic underachievement, alongside other psychological, educational, and contextual variables. The results also suggest that interventions focusing solely on emotional regulation may be insufficient, emphasizing the need to prioritize self-awareness alongside emotional skills. Educators, school counselors, and psychologists can incorporate training programs that help students monitor and regulate their smartphone use, set academic goals, and maintain focus during learning activities. Furthermore, these findings can inform curriculum design and school policies, promoting the integration of mindfulness and self-regulation strategies to enhance academic engagement, support mental well-being, and improve overall student performance.

### Limitations and future directions

The present study has several limitations. The use of a convenience sample of Bangladeshi adolescent and young adult students limits the generalizability of the findings to the broader youth and adult populations. Future research should recruit more diverse samples, including participants from different regions, educational backgrounds, and cultural contexts, to improve generalizability. The cross-sectional design restricts causal interpretations, highlighting the need for longitudinal or experimental studies to establish temporal and causal relationships among smartphone addiction, mindful attention, and academic outcomes. Data were collected through self-reported measures, which may be influenced by social desirability or recall biases; future research should incorporate objective assessments such as digital usage tracking or academic records to enhance validity. Although classroom mindful attention was examined as an associating factor, other factors such as parental control, motivation, and sleep quality—which research indicates can be negatively impacted by smartphone addiction and subsequently affect academic performance [131,151]—were not investigated. Future studies should examine the roles of these additional mediating and moderating factors. Additionally, smartphone addiction was treated as a single construct, without differentiating between types of use, such as social media, gaming, or educational activities [76,152]. Future research should explore how specific patterns of smartphone use differentially influence mindful attention and academic underperformance. Finally, both EFA and CFA were conducted using the same dataset rather than independent exploratory and confirmatory subsamples. Although the sample size was adequate for psychometric analyses, using the same sample for both exploratory and confirmatory procedures may have capitalized on chance and resulted in somewhat optimistic estimates of the factor structure and model fit. Future studies are encouraged to replicat these findings using independent samples or split-sample cross-validation to provide stronger evidence for the stability and generalizability of the measurement models.
