# Supplementary material for: Bangla validation of the smartphone addiction scale and the academic underachievement scale: The associative role of classroom mindful attention in the relationship between smartphone addiction and underachievement among adolescents and young adults
Source: PLoS One. 2026 Jul 16;21(7):e0353865. doi: 10.1371/journal.pone.0353865 (PMC13374972; doi:10.1371/journal.pone.0353865)
Supplement: Attachment — Submitted filename: Review Report_PONE-D-25-56548.pdf [file pone.0353865.s001.pdf]

Classroom Mindful Attention Mediating the Relationship between Smartphone Addiction and Academic Underperformance of Adolescents and Young Adults

Dear authors,

I am thankful for the opportunity PLOS One has given me to read and review your work as a researcher and as a reader. The Bangladeshi-specific findings add new knowledge to academia, which I personally admire, as more evidence is needed from South Asian contexts regarding data-collection instrument validation. However, while reading your work, certain concerns emerged. Hence, I will point them out so you can help readers better understand your work with greater clarity than in the current version. If my comments make sense, please address them because these comments will increase the readability and rigour of your paper.

Title:

1. I do not have any significant concerns with the title. But most of your methods were described by giving greater attention to the validation of data collection tools than to the mediation analysis. Hence, it would be better if you could revisit your title.

Abstract:

2. The content covered the necessary sections. However, readers will benefit if you report the results of the psychometric validation and the mediation analysis with relevant statistics. Because you mentioned that instrument validation was one of your aims.
    a. You mentioned an “excellent internal consistency” but did not report Cronbach’s α or reliability coefficients.
    b. You mentioned that you “confirmed the predefined factor structures” but did not report CFA fit indices.
    c. You mentioned a “partially mediated” but did not report mediation coefficients, indirect effects, or significance levels.
3. I noticed that there is a conceptual inconsistency with the wording. i.e., “Smartphone addiction resulted in lower academic achievement…” Shouldn’t this be ‘perceived’ … academic achievement?
    a. Because this study was claimed to be a cross-sectional one, could you reconsider your wording that suggests the ‘causality’?
4. Given the sample size and the results, it would be better to reconsider your conclusion remarks in a neutral tone. 😊

Introduction:

5. Your introduction satisfactorily established background information on the global smartphone prevalence, its relevance for adolescents, and its role in attention and mindfulness literature by hinting at a research gap regarding mediation effects.
    a. Did you build the mediation hypothesis based on any theoretical framework? I suggest that you capture the relevant theoretical explanations to validate the hypothesis that you are interested in.

6. Line 154: What does ITC stand for? Will it help readers outside the field if I mention the full version of this abbreviated version?
7. Line 112 onwards: Please revisit the numbering of your aims. Is aim number 3 missing?
    a. What you mentioned as the aim in the abstract is different (conceptually the same) from what is mentioned here. Can these aims be treated as specific objectives, given that you mentioned a broader perspective before? (Sometimes it is just wordplay. 😊 But we need those terms accurately to make sure that we present this paper according to the Western school of writing. 😊)

Methods:

It is a strong point that you have a considerable sample size for this validation study and the mediation analysis. Also, you have used several advanced psychometric methods.

However, I have several major concerns about the methodological explanations of your study.

8. You mentioned that the sample was recruited based on convenience. However, you did not mention how you plan to address the limitations of convenience sampling. Could you add this information to the methods section?
9. When data are collected across two different channels, such as online and physical, there can be a difference in the responses. Could you tell the readers how you analysed the 'mode effects' in your scenario?
10. The age groups (12-18 and 19–27-year-olds) are substantially different in smartphone usage, academic context and their attention demands 😊 (Even within the two cohorts, based on their developmental stages, those aforesaid facts are influencing them in different ways). Therefore, why did you decide to analyse all these age groups together in the mediation analysis? You may provide a sound rationale for this decision.
11. Please correct me if I am wrong. The PAUS scale was originally designed for adults. But your study includes 12-year-olds. Is it recommended to use this scale among minors?
12. You have thoroughly explained the methods for psychometric validations. However, there is no adequate information on the mediation methodology. Please briefly explain in the paper your missing assumption tests, bootstrapping procedure, estimation method, etc.
    a. Could you clearly specify the mediation method?
    b. Also, about the missing assumption tests.
    c. Concerns on age group confounding?
13. This comment is based on what I read. Your mediation mode is more like:

Smartphone Addiction (X) → Mindful Attention (M) → Academic Underperformance (Y)

If this is the case, based on cross-sectional data, it suggests that your model can only show statistical association, not mediation in a causal sense. The reason is that cross-sectional data measure all variables at the same time, and such data do not provide evidence that X occurs

before M, or that M occurs before Y. For true mediation, many researchers use longitudinal data or temporal measurement waves. Therefore, it is better if the authors consider the mode mentioned in your paper as a path model/ statistical mediation/ indirect association.

Results:

14. It is praiseworthy that the analysis results are generally consistent. However, in the mediation section, you mentioned "$\beta = 0.83$, 95% CI [0.37, 0.41]". Can the beta value be outside the CI? This can be a major reporting error. Please revisit.
15. The GPA correlation was indicated to be weak ($r = -0.08$). But your discussion section interpreted that as meaningful. Is there any specific reason behind that?

Discussion:

The authors satisfactorily connect findings to previous research. But the discussion still shows redundancy (several ideas and findings are repeated across multiple paragraphs without adding new interpretation or theoretical development). The limitations section is reasonably honest. However, there are some concerns again.

16. Well, this cross-sectional study hints at causal claims through the discussion, which is not wise (given the study design). For example, claims such as "smartphone addiction impairs academic success…" suggest causal claims.
17. As I mentioned under the observations for the Introduction section, the discussion section demonstrated the lack of integration with relevant theories.
18. In addition to the limitations mentioned, the authors may discuss the self-report questionnaires (missing objective data), measurement validation and hypothesis testing done in the same sample, age heterogeneity and any measurement bias.

Other Comments:

19. Thank you for your hard work. 😊 Overall, my major concern is that your mediation analysis is based on cross-sectional data. Because smartphone addiction, classroom mindful attention, and academic underachievement were measured at the same time point, the temporal ordering required for causal mediation cannot be established. As a result, the analysis only demonstrates statistical associations rather than a true mediating process. The manuscript nevertheless interprets the findings causally (e.g., suggesting that smartphone addiction impairs academic success through reduced self-awareness). You should either (1) substantially temper causal language and frame the results as associative pathways, or (2) justify the mediation framework more carefully, given the cross-sectional design.
