# Supplementary material for: Bangla validation of the smartphone addiction scale and the academic underachievement scale: The associative role of classroom mindful attention in the relationship between smartphone addiction and underachievement among adolescents and young adults
Source: PLoS One. 2026 Jul 16;21(7):e0353865. doi: 10.1371/journal.pone.0353865 (PMC13374972; doi:10.1371/journal.pone.0353865)
Supplement: Attachment — Submitted filename: Review Report_PLOSONE_PONE-D-25-56548_R1.pdf [file pone.0353865.s003.pdf]

Bangla Validation of the Smartphone Addiction Scale and the Academic Underachievement Scale: The Associative Role of Classroom Mindful Attention in the Relationship Between Smartphone Addiction and Underachievement Among Adolescents and Young Adults

Dear authors,

I am thankful for the opportunity that PLOS One has given me to read and review your revised work as a researcher and as a reader. The revised manuscript seemed substantially improved. The paper seemed to have addressed the editor's or previous original reviewers' main concerns regarding the unclear contribution and have revised the manuscript extensively. The psychometric validation component is strong and likely constitutes the primary contribution of the study. Yet there seemed to be some remaining issues, mostly related to clarification, interpretation, and reporting rather than fundamental methodological flaws.

1. The manuscript validated both scales using the same dataset of 712 participants. There is no indication that the sample was split into exploratory and confirmatory subsamples. Could you acknowledge that approach as a limitation?
2. Although you have appropriately replaced mediation language with "indirect association," you still used statements such as "higher smartphone addiction was associated with lower classroom self-awareness, which in turn was associated with higher perceived academic underachievement". This wording is much improved, but some parts of the discussion section still drifted toward mechanistic explanations that cannot be established using cross-sectional data. Could you revisit?
3. You have acknowledged convenience sampling, but the statement "sampling diversity, thereby supporting the normality assumptions for the present sample" is still problematic because sampling diversity does not establish normality.
4. Your manuscript states "consent was additionally obtained from their parents or teachers (cases where parents were not available)." I recommend providing further clarification regarding the ethical justification for teacher consent.
5. Ver small indirect effects such as B = 0.014 and B = 0.020 should be more explicitly addressed in relation to their practical significance. In other words, the indirect associations reported in your study are statistically significant. However, the magnitude of the indirect effects appears relatively small (B = 0.014 and B = 0.020). It is better to provide a brief discussion of the practical significance of these findings. While statistical significance demonstrates the presence of an association, readers would benefit from understanding the extent to which classroom mindful attention may contribute meaningfully to the relationship between smartphone addiction and perceived academic underachievement in real-world educational settings.
6. The reported reliability coefficients are very high for both scales. The authors may consider briefly commenting on whether these values reflect strong construct

homogeneity and whether any concerns regarding potential item redundancy should be considered in future research.

Overall, the manuscript makes a useful contribution to the literature on smartphone addiction, academic underachievement, and classroom mindful attention among Bangladeshi adolescents and young adults. Thank you.
