# Supplementary material for: Bangla validation of the smartphone addiction scale and the academic underachievement scale: The associative role of classroom mindful attention in the relationship between smartphone addiction and underachievement among adolescents and young adults
Source: PLoS One. 2026 Jul 16;21(7):e0353865. doi: 10.1371/journal.pone.0353865 (PMC13374972; doi:10.1371/journal.pone.0353865)
Supplement: Attachment — Submitted filename: Responses to Reviewers.docx [file pone.0353865.s002.docx]

**Editorial Comments**

I have now received two reports from expert reviews. They both find the paper interesting but also provide many major comments that deal with almost all parts of the manuscript. Based on my own reading, I also find the paper interesting but see two key flaws: the contribution is unclear and the mediation model is entirely unexplained whereas the paper seems to deal with mediation. The authors should thoroughly rewrite the manuscript to make it more clear.

**[Authors]** We would like to express our sincere gratitude to the Editor. Based on the Editor’s invaluable comments addressing two key flaws, we have thoroughly revised/rewritten the manuscript to make the points clear and coherent.

**Responses to Reviewer #1 Comments to the Authors**

**[Comments]** Thank you for the opportunity to review this manuscript, which examines Classroom Mindful Attention Mediating the Relationship between Smartphone Addiction and Academic Underperformance of Adolescents and Young Adults. The research topic of this manuscript has inherent theoretical significance and practical relevance. It is aligned with current priorities in science and education and addresses educational challenges, which is an advantage worth acknowledging. The abstract is clearly written, the study appears complete, and the sample size is relatively large. However, after reviewing, several areas in the manuscript were identified that require further review and editing.

**[Authors]** We would like to express our sincere gratitude for the careful evaluation of the manuscript and for recognizing its theoretical significance, practical relevance, and alignment with current priorities in science and education. We also appreciate the positive remarks regarding the clarity of the abstract, the completeness of the study, and the adequacy of the sample size. Your constructive feedback highlighting areas that require further review and improvement have appreciably accepted. We have carefully addressed all the concerns raised and revised the manuscript accordingly to enhance clarity, methodological rigor, and overall presentation. We believe that the revisions have significantly improved the quality and readability of the manuscript.

**[Comments-1]** The title is not well presented. It would be better to say: “The Impact of Smartphone Addiction and Academic Underperformance: The Mediating Role of Mindful Attention in the Classroom among Adolescents and Young Adults.” Hence, it would be better if you could revisit your title.

**[Authors]** We sincerely appreciate your suggestion regarding the title. We have revised it as recommended to improve clarity and accurately reflect the study’s focus. For your kind note, the Reviewer 2 has raised concerns that most of our methods have been described by giving greater attention to the validation of data collection tools than to the mediation analysis. Moreover, Reviewer 2 has noted that due to the cross-sectional nature of the study, causal explanations throughout the manuscript including the results section should be replaced by associative relationships through indirect pathways instead of mediation. Taken together, we have rephrased the title as ‘Bangla Validation of the Smartphone Addiction Scale and the Academic Underachievement Scale: The Associative Role of Classroom Mindful Attention in the Relationship Between Smartphone Addiction and Underachievement Among Adolescents and Young Adults’.

**[Comments-2]** At the beginning of the theoretical introduction, the authors introduce us to the smartphone market and its importance and confirm that mobile technologies have changed the paradigm of life. Introduce the readers of the paper to the situation in Bangladesh. Offer insight into the environment by briefly describing the educational context of learning in Bangladesh, the organization of schools and faculties, teaching methods and the rules for using phones, i.e. their accessibility in the educational process. For example: How much time do students spend on their phones? Is their use allowed at school or at the faculty? If there are certain rules, state them and introduce the readers of the paper. Compare the statements with other educational systems. This is an important aspect when considering the impact of technology on education, as well as on social life.

**[Authors]** Thank you for this valuable suggestion. We agree that the initial introduction did not sufficiently contextualize the educational environment in Bangladesh. In the revised manuscript, we have strengthened this section by adding a clearer description of the Bangladeshi educational context, including the structure of schools and higher education institutions, patterns of smartphone use among students, and general norms regarding mobile phone accessibility in academic settings. In addition, we have incorporated a brief comparison with other educational systems to better highlight differences in the pedagogical integration and regulation of mobile technologies. These revisions improve the contextual grounding of the study and clarify the relevance of smartphone use within both national and global educational settings.

**[Comments-3]** The main focus of the manuscript is on adolescents and young adults with their tendency to overuse mobile phones, the authors cite mobile phone addiction as a negative consequence of their overuse. Familiarize readers with the views of the World Health Organization. Also, what about parental control and student self-control?

**[Authors]** Thank you for this important comment. We have revised the manuscript to include the perspective of the World Health Organization, noting that while smartphone addiction is not officially classified as a separate disorder, behavioral addictions such as gaming disorder are recognized. We also expanded the discussion on parental guidance and student self-control. The revised version explains how supportive but firm parenting (authoritative parenting) can help reduce problematic smartphone use. In addition, we highlighted the role of self-control in managing smartphone behavior. We included supporting evidence showing that students with low self-control are more likely to use smartphones excessively, respond quickly to notifications, and engage in procrastination. These revisions help clarify that smartphone addiction is closely related to the self-regulatory mechanisms of an individual.

**[Comments-4]** Help readers reinforce the theoretical part of the paper related to attentional awareness and emotional regulation. Explain why these two components are important in an educational context. Can you help readers more clearly connect and understand the findings of studies linking attention and emotional regulation to learning and academic achievement with the psychological use of broader psychological theoretical approaches that explain learning and behavior? The authors could make their work on attention in the workplace, mobile phone addiction, and academic success more theoretically grounded, by citing psychological approaches that can explain the mediating role of attention in students: For example, Cognitive Theories of Attention, which would more clearly show how digital stimuli affect students' focus, then, Self-regulation Theory, which would provide insight into different abilities of attention control and provide insight into the orientation towards the workplace, but also Socio-Cultural Theory that indicates how the smartphone is a "tool" that changes interaction and learning, etc. It is important to emphasize the mediation in the relationship between addiction and academic success, but also for the authors to support this insight with relevant research. This omission limits the theoretical depth and academic contribution of the study.

**[Authors]** Thanks a lot for this insightful suggestion. We have strengthened the theoretical foundation of the study by expanding the discussion on attentional control, emotional regulation, and mindfulness in the educational context. The revised manuscript now explains how classroom mindful attention helps students stay focused, manage their emotions, and engage more effectively in learning activities. We also incorporated relevant theoretical perspectives, especially self-regulation theory, to explain how mindfulness improves attention control and reduces impulsive behaviors such as excessive smartphone use. In addition, we clarified how poor attentional control and weak emotional regulation can lead to academic disengagement and problematic technology use. While our revision mainly focuses on self-regulation theory, we acknowledge that other perspectives, such as cognitive theories of attention and socio-cultural theory, could further strengthen the discussion.

**[Comments-5]** In the part of the paper that deals with the methodology, the sampling procedure of the respondents is not clearly described. How were adolescents and young adults selected, i.e. what criterion was used for the selection of educational institutions?

**[Authors]** Thank you for your valuable comments. We have revised the Methods section to clarify the sampling procedure. Specifically, we have added details regarding the selection of educational institutions, noting that they were chosen based on accessibility, willingness to participate, and administrative approval. We also clarified the participant recruitment process (e.g., classroom visits and announcements), the inclusion criteria (aged 12–27 years), and noted that parental consent was obtained for participants under 18 years of age. Within these institutions, students who were present at the time of data collection (offline) and provided informed consent were included in the study. These additions will improve transparency in the sampling process.

**[Comments-6]** Did the sample include schools and faculties with different levels of academic achievement of young people (high, medium or low)? How might this affect the obtained results? There is a lack of data on academic achievements and competencies related to the use of mobile phones of students and pupils included in the research. Presenting the research results according to the level of academic success of the respondents would contribute to a better understanding and interpretation of the obtained results.

**[Authors]** Thank you for these important queries. We examined differences across academic achievement levels (high, medium, and low) using one-way ANOVA. The results showed no significant differences in smartphone addiction [*F* (2, 709) = 1.47, *p* = .231], classroom mindful attention [*F* (2, 709) = 1.52, *p* = .219], or perceived academic underperformance [*F* (2, 709) = 1.56, *p* = .210], with very small effect sizes (η²p = 0.004). These findings suggest that academic achievement levels did not significantly influence the study variables in the present sample.

**[Comments-7]** The paper also states that the sample included students from science and non-science faculties. Do they belong to the same field or different fields?

**[Authors]** Sincere thanks for your queries. The sample included participants from different academic fields. The science group comprised faculties such as natural sciences and engineering, while the non-science group included social sciences, humanities, and business studies. Thus, these categories represent distinct academic domains under a broader term (faculty) rather than the same field. The combination of faculties was categorized as academic streams (science/non-science). This diversity was intended to improve the generalizability of the findings across academic backgrounds.

**[Comments-8]** The pilot study was conducted; however, the authors could have informed the readers about its more detailed results. For example, it is important to present to the readers the differences in relation to the main research, in relation to gender, faculty, marital status, etc.

**[Authors]** We sincerely thank the reviewer for this valuable suggestion. The pilot study was conducted to assess the clarity, reliability, and feasibility of the survey instruments before the main study. It involved a small subset of participants drawn from the same population as the main study. The pilot sample was representative of the main sample, with characteristics such as gender, age, academic discipline (science vs. non-science), and so on similar to those of the main study. These findings indicated that the instruments were appropriate for the main study and helped refine the questionnaires where necessary. We have now added these details to the Methods section to improve transparency and provide readers with a clearer understanding of the pilot study outcomes.

**[Comments-9]** The measurement instruments used in the study are well characterized. The data collected by the instruments refer to self-assessments of young adults and students, which needs to be emphasized. In the statistical analysis, the authors can better present the results of the work if they stratify them and present them by gender, faculty, and age.

**[Authors]** Thank you for this valuable comment. In response, we have clarified that the study is based on self-reported data collected from adolescents and young adults using validated self-assessment instruments. We also conducted stratified analyses by gender, age group, and study field to better present the results as suggested. The independent samples t-tests showed no significant differences across gender, age groups, or study fields for smartphone addiction (SAS), classroom mindful attention (CMARS), or perceived academic underperformance (PAUS) (all p > .05). These findings indicate that the study variables are not significantly influenced by these demographic factors in the present sample, supporting their use in subsequent analyses.

**[Comments-10]** The manuscript acknowledges the problems; however, it is necessary to provide clearer details regarding validation.

**[Authors]** We sincerely thank the reviewer for this important suggestion. We have revised the manuscript to provide clearer and more detailed information regarding the validation process. Specifically, we have explicitly described the multiple stages of validation, including internal consistency reliability, construct validity through exploratory and confirmatory factor analyses, convergent validity, and item-level evaluation using Item Response Theory (IRT) models. These additions clarify how the psychometric properties of the instruments were established and improve the transparency and rigor of the methodology.

**[Comments-11]** In addition, the suggestions for future research are too general. Supplementing them with more detailed reflections on the limitations and practical recommendations for improvement will better demonstrate the critical engagement of the authors in their work.

**[Authors]** Thank you for this valuable suggestion. In response, we have strengthened the limitations and future research section by adding a more specific methodological reflection. We now acknowledge that both psychometric validation and hypothesis testing were conducted within the same sample, which may lead to inflated estimates of reliability and model fit. Accordingly, we recommend that future studies use independent samples for scale validation and structural model testing to enhance robustness and generalizability.

**Responses to Reviewer #2 Comments to the Authors**

**[Comments]** The manuscript addresses an important research question and includes valuable psychometric analyses, but substantial revisions are needed to correct statistical reporting errors, strengthen methodological transparency, and improve how you reported the results in the discussion section. My comments are attached in a separate file. Hope the comments will support you.

Classroom Mindful Attention Mediating the Relationship between Smartphone Addiction and Academic Underperformance of Adolescents and Young Adults Dear authors, I am thankful for the opportunity PLOS One has given me to read and review your work as a researcher and as a reader. The Bangladeshi-specific findings add new knowledge to academia, which I personally admire, as more evidence is needed from South Asian contexts regarding data-collection instrument validation. However, while reading your work, certain concerns emerged. Hence, I will point them out so you can help readers better understand your work with greater clarity than in the current version. If my comments make sense, please address them because these comments will increase the readability and rigor of your paper.

**[Authors]** We sincerely express our thanks and gratitude to the reviewer for taking the time to read and provide thoughtful feedback on our manuscript. We greatly appreciate your recognition of the importance of our research question and the contribution of Bangladeshi-specific findings to the literature. Your comments regarding statistical reporting, methodological transparency, and clarity in the discussion are invaluable. We have carefully considered all your suggestions and have **revised the manuscript accordingly** to correct statistical reporting errors, enhance methodological details, and improve the presentation and interpretation of the results in the discussion section. We believe these revisions have strengthened the rigor, readability, and overall quality of the manuscript. We are grateful for your constructive guidance, which has helped us improve the paper significantly.

**[Comments-1]** Title: I do not have any significant concerns with the title. But most of your methods were described by giving greater attention to the validation of data collection tools than to the mediation analysis. Hence, it would be better if you could revisit your title.

**[Authors]** Thank you for your insightful comment. We acknowledge that a substantial portion of the Methods and Results sections focuses on the validation of the data collection tools to ensure reliability and accuracy, which may have drawn attention away from the mediation analysis. However, the primary aim of our study was to examine the mediating role of mindful attention between smartphone addiction and academic underperformance. To clarify this focus, we have revised the title (following Reviewer 1’s suggestion) to emphasize the mediation analysis while retaining the necessary methodological rigor. We believe this revised title now better represents the study’s main objectives and findings.

**Abstract:**

**[Comments-2]** The content covered the necessary sections. However, readers will benefit if you report the results of the psychometric validation and the mediation analysis with relevant statistics. Because you mentioned that instrument validation was one of your aims.

**[Authors]** We thank the reviewer for this important suggestion. We have revised the Abstract to include key statistical results from both the psychometric validation (e.g., reliability coefficients and CFA fit indices) and the mediation analysis (e.g., indirect effects and significance levels). This ensures that the abstract better reflects the study’s objectives and findings.

**[Comments-3]** You mentioned an “excellent internal consistency” but did not report Cronbach’s α or reliability coefficients.

**[Authors]** We agree with the reviewer and have now included Cronbach’s alpha (α) and reliability coefficients in the Abstract to support the statement regarding internal consistency.

**[Comments-4]** You mentioned that you “confirmed the predefined factor structures” but did not report CFA fit indices.

**[Authors]** We appreciate this observation. Relevant CFA fit indices (e.g., CFI, TLI, RMSEA, SRMR) have now been included in the Abstract to provide evidence supporting the factor structure.

**[Comments-5]** You mentioned a “partially mediated” but did not report mediation coefficients, indirect effects, or significance levels.

**[Authors]** We thank the reviewer for this suggestion. We have revised the Abstract to include key mediation results reflecting associative relationships through indirect pathways for a cross sectional study, supporting the statement regarding associative relationships.

**[Comments-6]** I noticed that there is a conceptual inconsistency with the wording. i.e., “Smartphone addiction resulted in lower academic achievement…” Shouldn’t this be ‘perceived’ … academic achievement? a. Because this study was claimed to be a cross-sectional one, could you reconsider your wording that suggests the ‘causality’?

**[Authors]** Thank you for your invaluable suggestions. We would like to clarify that academic achievement in our study was measured objectively using Grade point averages from the most recent exam, whereas perceived academic underachievement was assessed separately via the self-assessment tool PAUS. Therefore, the original wording implying that smartphone addiction “resulted in lower academic achievement” could be misleading. We have revised the text to accurately reflect the findings: smartphone addiction was associated with lower academic performance (GPAs) and reduced classroom mindful attention (CMARS). Additionally, perceived academic underachievement (PAUS) was inversely related to both GPAs and classroom mindful attention, indicating that students who feel underachieving than their abilities through PAUS tend to have slightly lower academic performance (GPAs) and reduced mindful attention in classroom. We have also revised the wording in the Abstract to avoid causal language, replacing phrases such as ‘resulted in’ with ‘was associated with’ to better reflect the cross-sectional nature of the study. This revision ensures conceptual clarity and accurately distinguishes between actual academic performance (GPAs) and perceived underachievement (PAUS).

**[Comments-7]** Given the sample size and the results, it would be better to reconsider your conclusion remarks in a neutral tone.

**[Authors]** Thank you for your suggestion. We have adjusted the conclusion to a more neutral tone, indicating that these findings highlight the associative role of classroom self-awareness in the relationship between smartphone addiction and perceived academic underachievement and underscore the potential value of mindfulness-based interventions in educational settings.

**Introduction:**

**[Comments-8]** Your introduction satisfactorily established background information on the global smartphone prevalence, its relevance for adolescents, and its role in attention and mindfulness literature by hinting at a research gap regarding mediation effects.

Did you build the mediation hypothesis based on any theoretical framework? I suggest that you capture the relevant theoretical explanations to validate the hypothesis that you are interested in. Page 1 of 3 Classroom Mindful Attention Mediating the Relationship between Smartphone Addiction and Academic Underperformance of Adolescents and Young Adults

**[Authors]** Thank you for this important observation. We have clarified the theoretical basis of our mediation hypothesis by grounding it in self-regulation theory. The revised manuscript explains that mindfulness improves attentional control, emotional regulation, and self-awareness, which are key mechanisms linking smartphone use behaviors to academic outcomes. Specifically, we explain that excessive smartphone use can disrupt attention and self-regulation, which may lead to poorer academic performance. In contrast, classroom mindful attention can help students regain focus and stay engaged in learning. This provides a clear theoretical explanation for why classroom mindful attention plays an indirect associative role in the relationship between smartphone addiction and perceived academic underachievement.

**[Comments-9]** Line 154: What does ITC stand for? Will it help readers outside the field if I mention the full version of this abbreviated version?

**[Authors]** We sincerely thank the reviewer for this suggestion. We have now expanded the abbreviation “ITC” to its full form, **International Test Commission**, to improve clarity for readers who may not be familiar with the abbreviation. The manuscript has been updated accordingly.

**[Comments-10]** Line 112 onwards: Please revisit the numbering of your aims. Is aim number 3 missing? a. What you mentioned as the aim in the abstract is different (conceptually the same) from what is mentioned here. Can these aims be treated as specific objectives, given that you mentioned a broader perspective before? (Sometimes it is just wordplay. But we need those terms accurately to make sure that we present this paper according to the Western school of writing.)

**[Authors]** Thank you for this careful observation and suggestions regarding the clarity and numbering of the study aims. We acknowledge that there was an inconsistency in the original manuscript, where one objective was not clearly numbered, and the wording between the abstract and the objectives required better alignment. In the revised manuscript, we have carefully corrected the numbering of all objectives and ensured consistency between the abstract and the listed aims. We have also revised the abstract to ensure it accurately reflects all stated objectives in a consistent and coherent manner. These revisions improve clarity and ensure full alignment between the abstract and the methodological aims of the study.

**Methods:**

It is a strong point that you have a considerable sample size for this validation study and the mediation analysis. Also, you have used several advanced psychometric methods. However, I have several major concerns about the methodological explanations of your study.

**[Comments-11]** You mentioned that the sample was recruited based on convenience. However, you did not mention how you plan to address the limitations of convenience sampling. Could you add this information to the methods section?

**[Authors]** We appreciate this important suggestion. We have now briefly addressed the limitations of convenience sampling in the Methods section, acknowledging potential selection bias and limited generalizability. To mitigate potential sampling bias, data were collected from multiple educational institutions (schools, colleges, and universities) across diverse demographic backgrounds, including variations in age, gender, academic level, and socioeconomic status. Although the sample may not be fully representative of the broader population, efforts were made to enhance its diversity and the data met the normality assumptions through skewness and kurtosis thresholds.

**[Comments-12]** When data are collected across two different channels, such as online and physical, there can be a difference in the responses. Could you tell the readers how you analyzed the ‘mode effects’ in your scenario?

**[Authors]** Thank you for this important comment regarding potential mode effects arising from data collection through both online and offline channels. In response, we have conducted a multi-group confirmatory factor analysis to examine measurement invariance of the SAS-SV and PAUS across data collection modes along with age and gender. The results supported measurement invariance across data collection modes, as changes in model fit indices were within recommended thresholds (ΔCFI ≤ 0.010; ΔRMSEA ≤ 0.015). This indicates that configural, metric, and scalar invariance were satisfied, suggesting that participants across online and offline modes interpreted and responded to the items in a **consistent manner**. Accordingly, we did not observe evidence of systematic mode effects that would bias the construct measurement, and therefore the data collected via different modes were considered suitable for combined analysis and would be generalizable for further cross-cultural validations.

**[Comments-13]** The age groups (12-18 and 19–27-year-olds) are substantially different in smartphone usage, academic context and their attention demands (Even within the two cohorts, based on their developmental stages, those aforesaid d facts are influencing them in different ways). Therefore, why did you decide to analyses all these age groups together in the mediation analysis? You may provide a sound rationale for this decision.

**[Authors]** Thank you for this insightful observation regarding the heterogeneity of the age groups. We agree that adolescents (12–18 years) and young adults (19–27 years) differ in terms of developmental stage, smartphone usage patterns, academic demands, and attentional capacities, which may influence the relationships examined in this study. In the initial analysis, we used the combined sample to examine the overall relationships among smartphone addiction, classroom mindful attention, and perceived academic underachievement. This approach was based on our objective to test the proposed pathway model in the full sample. In addition, measurement invariance was established across age groups, indicating that the constructs were comparable and could be analyzed together.

In response to the reviewer’s suggestion, we have now conducted separate analyses for adolescents and young adults, while also retaining the combined sample results. This allowed us to better understand whether the relationships differ across developmental stages. The revised analyses reveal some notable differences between the groups. Specifically, the indirect association through classroom self-awareness was not statistically significant among adolescents but was significant among young adults and in the combined sample. In contrast, emotional regulation did not show a significant indirect association in any group. These findings suggest that the associative role of mindful attention, particularly self-awareness, may become more salient in later developmental stages when cognitive and self-regulatory capacities are fully developed. At the same time, the direct association between smartphone addiction and perceived academic underachievement remained consistently strong and significant across all groups, supporting the robustness of the primary relationship. Overall, by presenting both combined and group-specific analyses, the revised manuscript provides a more nuanced understanding of the relationships while maintaining the theoretical coherence of the model.

**[Comments-14]** Please correct me if I am wrong. The PAUS scale was originally designed for adults. But your study includes 12-year-olds. Is it recommended to use this scale among minors?

**[Authors]** We thank the reviewer for this important observation. The PAUS was originally developed for adults; however, in our study, we adapted it for use with adolescents aged 12 and above. Prior to the main study, we conducted a **pilot study** to ensure that the scale was **linguistically clear, culturally relevant, and comprehensible** for younger participants. Feedback from the pilot confirmed that the items were understandable and appropriate for adolescents, and minor modifications were made to enhance clarity without altering the original meaning. Therefore, we considered the PAUS suitable for use among participants aged 12 and older in the current study.

**[Comments-15]** You have thoroughly explained the methods for psychometric validations. However, there is no adequate information on the mediation methodology. Please briefly explain in the paper your missing assumption tests, bootstrapping procedure, estimation method, etc.

a. Could you clearly specify the mediation method?

b. Also, about the missing assumption tests.

c. Concerns on age group confounding?

**[Authors]** Thank you for this important suggestion. We have revised the manuscript to provide clearer and more detailed information regarding the mediation analysis.

**a) Specification of mediation method:** We have now explicitly stated that the mediation analysis was conducted using a **generalized linear model (GLM) mediation framework**. The mediation model was tested using full information maximum likelihood (FIML) estimation, and indirect effects were assessed using bootstrap percentile confidence intervals.

**(b) Missing assumption tests and bootstrapping procedure:** We thank the reviewer for this suggestion. We have revised the manuscript by clearly describing the mediation approach, including the use of FIML under MAR for missing data handling (Enders, 2010; Kaplan, 2009) and bootstrap percentile confidence intervals for testing indirect effects (Preacher & Hayes, 2008), improving methodological clarity and transparency.

**(c) Concerns on age group confounding:** We acknowledge the reviewer’s concern regarding potential confounding effects of age group differences. To address this, we conducted **separate statistical mediation for adolescents and young adults in addition to the combined sample**, allowing us to examine whether the indirect pathways differ across developmental stages. Furthermore, measurement invariance across age groups was established prior to analysis, supporting the comparability of constructs between groups. The mediation results showed similar patterns in the direct effects across groups, while some differences emerged in the indirect pathways, which have been clearly discussed in the revised manuscript.

**Comments-16]** This comment is based on what I read. Your mediation mode is more like: Smartphone Addiction (X) → Mindful Attention (M) → Academic Underperformance (Y) If this is the case, based on cross-sectional data, it suggests that your model can only show statistical association, not mediation in a causal sense. The reason is that cross-sectional data measure all variables at the same time, and such data do not provide evidence that X occurs Page 2 of 3 Classroom Mindful Attention Mediating the Relationship between Smartphone Addiction and Academic Underperformance of Adolescents and Young Adults before M, or that M occurs before Y. For true mediation, many researchers use longitudinal data or temporal measurement waves. Therefore, it is better if the authors consider the mode mentioned in your paper as a path model/ statistical mediation/ indirect association.

**[Authors]** Thank you for this insightful and important comment. We agree with the Reviewer that given the cross-sectional nature of our data, it is not possible to establish temporal precedence or causal mediation among the variables. In response, we have carefully revised the manuscript to clarify that our analysis reflects statistical associations rather than causal mediation. Specifically, we have replaced the causal language “mediates” with more appropriate terminology such as “indirect association” throughout the Abstract, Methods, Results, and Discussion sections. We have also explicitly acknowledged in the manuscript that cross-sectional data do not allow us to determine whether smartphone addiction precedes changes in mindful attention or academic outcomes. Accordingly, we now emphasize that the findings should be interpreted as preliminary evidence of associations that require confirmation through longitudinal or experimental research designs. We appreciate the reviewer’s suggestion, which has helped us improve the conceptual clarity and methodological rigor of our manuscript.

**Results:**

**[Comments-17]** It is praiseworthy that the analysis results are generally consistent. However, in the mediation section, you mentioned “β = 0.83, 95% CI [0.37, 0.41]”. Can the beta value be outside the CI? This can be a major reporting error. Please revisit.

**[Authors]** We thank the reviewer for this valuable observation. We agree that the way the results were previously reported may have led to confusion. In the original version, the β value was mistakenly reported alongside the confidence interval of the unstandardized estimate, which created the inconsistency. To address this issue, we have revised the manuscript to clearly report the unstandardized estimates (with their corresponding confidence intervals and p-values). This ensures accurate and transparent reporting in line with recommended statistical practices.

**[Comments-18]** The GPA correlation was indicated to be weak (r = -0.08). But your discussion section interpreted that as meaningful. Is there any specific reason behind that?

**[Authors]** We thank the reviewer for this valuable comment. We acknowledge that the original interpretation may have overstated the practical strength of the association between GPA, smartphone addiction, and perceived academic underachievement. In the revised manuscript, we have clarified that although the relationships are statistically significant, their magnitude is weak, and we have accordingly revised the discussion to reflect a more cautious and accurate interpretation of the findings.

**[Comments-19]** **Discussion:** The authors satisfactorily connect findings to previous research. But the discussion still shows redundancy (several ideas and findings are repeated across multiple paragraphs without adding new interpretation or theoretical development). The limitations section is reasonably honest. However, there are some concerns again.

**[Authors]** We thank the reviewer for this helpful comment. We have revised the discussion by removing redundant content and improving coherence. We also strengthened the mediation-related interpretation by incorporating relevant theoretical perspectives and clarifying the GPA-related findings. The limitations section has also been refined for clarity and alignment with the study.

**[Comments-20]** Well, this cross-sectional study hints at causal claims through the discussion, which is not wise (given the study design). For example, claims such as “smartphone addiction impairs academic success…” suggest causal claims.

**[Authors]** Thank you for this important comment. We agree that causal language is not appropriate for a cross-sectional study. In response, we have revised the manuscript to temper all causal statements and instead frame the findings as associations or indirect associations. Specifically, statements suggesting that smartphone addiction “impairs academic success” have been reworded to reflect associative relationships.

**[Comments-21]** As I mentioned under the observations for the Introduction section, the discussion section demonstrated the lack of integration with relevant theories.

**[Authors]** We thank the reviewer for this important observation. We acknowledge that the initial version of the discussion did not sufficiently integrate relevant theoretical frameworks. In the revised manuscript, we have strengthened the theoretical integration by explicitly linking the findings to appropriate theories, particularly in the interpretation of key results, including the associative pathways. This revision enhances the conceptual grounding and overall coherence of the discussion section.

**[Comments-22]** In addition to the limitations mentioned, the authors may discuss the self-report questionnaires (missing objective data), measurement validation, and hypothesis testing done in the same sample, age heterogeneity, and any measurement bias.

**[Authors]** Thank you for this valuable comment. As noted in the manuscript, we have already addressed the limitations related to self-reported measures and their potential biases. In addition, we have clarified that the psychometric validation of the instruments and the hypothesis testing were conducted within the same sample, which may inflate estimates of reliability and model fit. We now recommend that future studies use independent samples for validation and structural analyses. These clarifications have been incorporated into the Limitations section to provide a more accurate interpretation of our findings.

**Other Comments:**

**[Comments-23]** Thank you for your hard work. Overall, my major concern is that your mediation analysis is based on cross-sectional data. Because smartphone addiction, classroom mindful attention, and academic underachievement were measured at the same time point, the temporal ordering required for causal mediation cannot be established. As a result, the analysis only demonstrates statistical associations rather than a true mediating process. The manuscript nevertheless interprets the findings causally (e.g., suggesting that smartphone addiction impairs academic success through reduced self-awareness).

You should either

(1) substantially temper causal language and frame the results as associative pathways,

or (2) justify the mediation framework more carefully, given the cross-sectional design.

**[Authors]** Thank you so much for your invaluable suggestions. We agree that the cross-sectional design does not allow for establishing temporal order or causal mediation. In response, we have substantially tempered the causal language throughout the manuscript. Specifically, we have reframed our analysis as examining indirect (associative) pathways rather than causal mediation, and we have replaced terms such as “mediates” with more appropriate wording (e.g., “indirect association” or “pathway”) across the Abstract, Methods, Results, and Discussion sections. We have also clarified that the results reflect statistical associations and have interpreted them with cautions. We appreciate the reviewer’s valuable comments, which has helped improve the clarity and rigor of our manuscript.

Thank you
