# Supplementary material for: Bangla validation of the smartphone addiction scale and the academic underachievement scale: The associative role of classroom mindful attention in the relationship between smartphone addiction and underachievement among adolescents and young adults
Source: PLoS One. 2026 Jul 16;21(7):e0353865. doi: 10.1371/journal.pone.0353865 (PMC13374972; doi:10.1371/journal.pone.0353865)
Supplement: Peer review history

# Peer review: Decision Letter 0

**DOI:** 10.1371/journal.pone.0353865.r001

**Authors:** Deni Mazrekaj

9 Mar 2026

PONE-D-25-56548

Classroom Mindful Attention Mediating the Relationship between Smartphone Addiction and Academic Underperformance of Adolescents and Young Adults

PLOS One

Dear Dr. Shahrier,

Thank you for submitting your manuscript to PLOS ONE. After careful consideration, we feel that it has merit but does not fully meet PLOS ONE’s publication criteria as it currently stands. Therefore, we invite you to submit a revised version of the manuscript that addresses the points raised during the review process.

Please submit your revised manuscript by Apr 23 2026 11:59PM. If you will need more time than this to complete your revisions, please reply to this message or contact the journal office at plosone@plos.org. When you're ready to submit your revision, log on to https://www.editorialmanager.com/pone/ and select the 'Submissions Needing Revision' folder to locate your manuscript file.

Please include the following items when submitting your revised manuscript:

- A letter that responds to each point raised by the academic editor and reviewer(s). You should upload this letter as a separate file labeled 'Response to Reviewers'.
- A marked-up copy of your manuscript that highlights changes made to the original version. You should upload this as a separate file labeled 'Revised Manuscript with Track Changes'.
- An unmarked version of your revised paper without tracked changes. You should upload this as a separate file labeled 'Manuscript'.

If you would like to make changes to your financial disclosure, please include your updated statement in your cover letter. Guidelines for resubmitting your figure files are available below the reviewer comments at the end of this letter.

If applicable, we recommend that you deposit your laboratory protocols in protocols.io to enhance the reproducibility of your results. Protocols.io assigns your protocol its own identifier (DOI) so that it can be cited independently in the future. For instructions see: https://journals.plos.org/plosone/s/submission-guidelines#loc-laboratory-protocols. Additionally, PLOS ONE offers an option for publishing peer-reviewed Lab Protocol articles, which describe protocols hosted on protocols.io. Read more information on sharing protocols at https://plos.org/protocols?utm_medium=editorial-email&utm_source=authorletters&utm_campaign=protocols.

We look forward to receiving your revised manuscript.

Kind regards,

Deni Mazrekaj

Academic Editor

PLOS One

Journal Requirements:

When submitting your revision, we need you to address these additional requirements.

1.Please ensure that your manuscript meets PLOS ONE's style requirements, including those for file naming. The PLOS ONE style templates can be found at

https://journals.plos.org/plosone/s/file?id=wjVg/PLOSOne_formatting_sample_main_body.pdf and

https://journals.plos.org/plosone/s/file?id=ba62/PLOSOne_formatting_sample_title_authors_affiliations.pdf

2. We note that you have indicated that there are restrictions to data sharing for this study. PLOS only allows data to be available upon request if there are legal or ethical restrictions on sharing data publicly. For more information on unacceptable data access restrictions, please see http://journals.plos.org/plosone/s/data-availability#loc-unacceptable-data-access-restrictions.

Before we proceed with your manuscript, please address the following prompts:

a) If there are ethical or legal restrictions on sharing a de-identified data set, please explain them in detail (e.g., data contain potentially identifying or sensitive patient information, data are owned by a third-party organization, etc.) and who has imposed them (e.g., a Research Ethics Committee or Institutional Review Board, etc.). Please also provide contact information for a data access committee, ethics committee, or other institutional body to which data requests may be sent.

b) If there are no restrictions, please upload the minimal anonymized data set necessary to replicate your study findings to a stable, public repository and provide us with the relevant URLs, DOIs, or accession numbers. For a list of recommended repositories, please see

https://journals.plos.org/plosone/s/recommended-repositories. You also have the option of uploading the data as Supporting Information files, but we would recommend depositing data directly to a data repository if possible.

We will update your Data Availability statement on your behalf to reflect the information you provide.

3. If the reviewer comments include a recommendation to cite specific previously published works, please review and evaluate these publications to determine whether they are relevant and should be cited. There is no requirement to cite these works unless the editor has indicated otherwise.

Additional Editor Comments:

I have now received two reports from expert reviews. They both find the paper interesting but also provide many major comments that deal with almost all parts of the manuscript. Based on my own reading, I also find the paper interesting but see two key flaws: the contribution is unclear and the mediation model is entirely unexplained whereas the paper seems to deal with mediation. The authors should thoroughly rewrite the manuscript to make it more clear.

[Note: HTML markup is below. Please do not edit.]

Reviewers' comments:

Reviewer's Responses to Questions

**Comments to the Author**

1. Is the manuscript technically sound, and do the data support the conclusions?

The manuscript must describe a technically sound piece of scientific research with data that supports the conclusions. Experiments must have been conducted rigorously, with appropriate controls, replication, and sample sizes. The conclusions must be drawn appropriately based on the data presented.

Reviewer #1: Yes

Reviewer #2: Partly

**********

2. Has the statistical analysis been performed appropriately and rigorously?

Reviewer #1: Yes

Reviewer #2: No

**********

3. Have the authors made all data underlying the findings in their manuscript fully available?

The PLOS Data policy requires authors to make all data underlying the findings described in their manuscript fully available without restriction, with rare exception (please refer to the Data Availability Statement in the manuscript PDF file). The data should be provided as part of the manuscript or its supporting information, or deposited to a public repository. For example, in addition to summary statistics, the data points behind means, medians and variance measures should be available. If there are restrictions on publicly sharing data—e.g. participant privacy or use of data from a third party—those must be specified.

Reviewer #1: Yes

Reviewer #2: No

**********

4. Is the manuscript presented in an intelligible fashion and written in standard English?

PLOS ONE does not copyedit accepted manuscripts, so the language in submitted articles must be clear, correct, and unambiguous. Any typographical or grammatical errors should be corrected at revision, so please note any specific errors here.

Reviewer #1: Yes

Reviewer #2: No

**********

5. Review Comments to the Author

Please use the space provided to explain your answers to the questions above. You may also include additional comments for the author, including concerns about dual publication, research ethics, or publication ethics. (Please upload your review as an attachment if it exceeds 20,000 characters)

Reviewer #1: Comments to the author

Thank you for the opportunity to review this manuscript, which examines Classroom Mindful Attention Mediating the Relationship between Smartphone Addiction and Academic Underperformance of Adolescents and Young Adults. The research topic of this manuscript has inherent theoretical significance and practical relevance. It is aligned with current priorities in science and education and addresses educational challenges, which is an advantage worth acknowledging. The abstract is clearly written, the study appears complete, and the sample size is relatively large. However, after reviewing, several areas in the manuscript were identified that require further review and editing.

My reviews are as follows:

1.The title is not well presented. It would be better to say: “The Impact of Smartphone Addiction and Academic Underperformance: The Mediating Role of Mindful Attention in the Classroom among Adolescents and Young Adults.”

2.At the beginning of the theoretical introduction, the authors introduce us to the smartphone market and its importance and confirm that mobile technologies have changed the paradigm of life. Introduce the readers of the paper to the situation in Bangladesh. Offer insight into the environment by briefly describing the educational context of learning in Bangladesh, the organization of schools and faculties, teaching methods and the rules for using phones, i.e. their accessibility in the educational process. For example: How much time do students spend on their phones? Is their use allowed at school or at the faculty? If there are certain rules, state them and introduce the readers of the paper. Compare the statements with other educational systems. This is an important aspect when considering the impact of technology on education, as well as on social life.

3.The main focus of the manuscript is on adolescents and young adults with their tendency to overuse mobile phones, the authors cite mobile phone addiction as a negative consequence of their overuse. Familiarize readers with the views of the World Health Organization. Also, what about parental control and student self-control?

4.Help readers reinforce the theoretical part of the paper related to attentional awareness and emotional regulation. Explain why these two components are important in an educational context. Can you help readers more clearly connect and understand the findings of studies linking attention and emotional regulation to learning and academic achievement with the psychological use of broader psychological theoretical approaches that explain learning and behavior? The authors could make their work on attention in the workplace, mobile phone addiction, and academic success more theoretically grounded, by citing psychological approaches that can explain the mediating role of attention in students: For example, Cognitive Theories of Attention, which would more clearly show how digital stimuli affect students' focus, then, Self-regulation Theory, which would provide insight into different abilities of attention control and provide insight into the orientation towards the workplace, but also Socio-Cultural Theory that indicates how the smartphone is a "tool" that changes interaction and learning, etc. It is

important to emphasize the mediation in the relationship between addiction and academic success, but also for the authors to support this insight with relevant research. This omission limits the theoretical depth and academic contribution of the study.

5.In the part of the paper that deals with the methodology, the sampling procedure of the respondents is not clearly described. How were adolescents and young adults selected, i.e. what criterion was used for the selection of educational institutions? Did the sample include schools and faculties with different levels of academic achievement of young people (high, medium or low)? How might this affect the obtained results? The paper also states that the sample included students from science and non-science faculties. Do they belong to the same field or different fields? There is a lack of data on academic achievements and competencies related to the use of mobile phones of students and pupils included in the research. Presenting the research results according to the level of academic success of the respondents would contribute to a better understanding and interpretation of the obtained results. The pilot study was conducted, however, the authors could have informed the readers about its more detailed results. For example, it is important to present to the readers the differences in relation to the main research, in relation to gender, faculty, marital status, etc.

6.The measurement instruments used in the study are well characterized. The data collected by the instruments refer to self-assessments of young adults and students, which needs to be emphasized. In the statistical analysis, the authors can better present the results of the work if they stratify them and present them by gender, faculty, and age.The manuscript acknowledges the problems, however, it is necessary to provide clearer details regarding validation. In addition, the suggestions for future research are too general. Supplementing them with more detailed reflections on the limitations and practical recommendations for improvement will better demonstrate the critical engagement of the authors in their work.

I sincerely hope that the authors will use these comments to thoroughly refine the study. When the above-mentioned issues are adequately addressed, the manuscript has the potential to provide a more valuable contribution to the field of science and technology education.

Sincerely,

reviewer

Reviewer #2: Dear Authors,

The manuscript addresses an important research question and includes valuable psychometric analyses, but substantial revisions are needed to correct statistical reporting errors, strengthen methodological transparency, and improve how you reported the results in the discussion section. My comments are attached in a separate file. Hope the comments will support you.

**********

6. PLOS authors have the option to publish the peer review history of their article (what does this mean?). If published, this will include your full peer review and any attached files.

If you choose “no”, your identity will remain anonymous but your review may still be made public.

**Do you want your identity to be public for this peer review?**  For information about this choice, including consent withdrawal, please see our Privacy Policy.

Reviewer #1: No

Reviewer #2: No

**********

[NOTE: If reviewer comments were submitted as an attachment file, they will be attached to this email and accessible via the submission site. Please log into your account, locate the manuscript record, and check for the action link "View Attachments". If this link does not appear, there are no attachment files.]

To ensure your figures meet our technical requirements, please review our figure guidelines: https://journals.plos.org/plosone/s/figures

You may also use PLOS’s free figure tool, NAAS, to help you prepare publication quality figures: https://journals.plos.org/plosone/s/figures#loc-tools-for-figure-preparation.

NAAS will assess whether your figures meet our technical requirements by comparing each figure against our figure specifications.

Attachment Submitted filename: Review Report_PONE-D-25-56548.pdf

# Author comment: Author response to Decision Letter 1

**DOI:** 10.1371/journal.pone.0353865.r002

21 Apr 2026

We would like to express our sincere gratitude to the Editor and the expert Reviewers. Based on the Editor’s and the Reviewers' invaluable comments addressing key flaws, we have thoroughly revised/rewritten the manuscript to make all the points clear and coherent. 'Responses to Reviewers' file attached here will provide further clarifications. Thank you.

Attachment Submitted filename: Responses to Reviewers.docx

# Peer review: Decision Letter 1

**DOI:** 10.1371/journal.pone.0353865.r003

**Authors:** Gladys Dzansi

1 Jul 2026

Bangla Validation of the Smartphone Addiction Scale and the Academic Underachievement Scale: The Associative Role of Classroom Mindful Attention in the Relationship Between Smartphone Addiction and  Underachievement Among Adolescents and Young Adults

PONE-D-25-56548R1

Dear Shahrier,

We’re pleased to inform you that your manuscript has been judged scientifically suitable for publication and will be formally accepted for publication once it meets all outstanding technical requirements.

Within one week, you’ll receive an e-mail detailing the required amendments. When these have been addressed, you’ll receive a formal acceptance letter and your manuscript will be scheduled for publication.

An invoice will be generated when your article is formally accepted. Please note, if your institution has a publishing partnership with PLOS and your article meets the relevant criteria, all or part of your publication costs will be covered. Please make sure your user information is up-to-date by logging into Editorial Manager at Editorial Manager® and clicking the ‘Update My Information' link at the top of the page. For questions related to billing, please contact billing support.

If your institution or institutions have a press office, please notify them about your upcoming paper to help maximize its impact. If they’ll be preparing press materials, please inform our press team as soon as possible -- no later than 48 hours after receiving the formal acceptance. Your manuscript will remain under strict press embargo until 2 pm Eastern Time on the date of publication. For more information, please contact onepress@plos.org.

Kind regards,

Gladys Dzansi, Ph.D, M.Phil, BA, RN

Academic Editor

PLOS One

Additional Editor Comments (optional):

Include the recommendations in the final submissions

Reviewers' comments:

Reviewer's Responses to Questions

**Comments to the Author**

1. If the authors have adequately addressed your comments raised in a previous round of review and you feel that this manuscript is now acceptable for publication, you may indicate that here to bypass the “Comments to the Author” section, enter your conflict of interest statement in the “Confidential to Editor” section, and submit your "Accept" recommendation.

Reviewer #1: All comments have been addressed

Reviewer #2: (No Response)

**********

2. Is the manuscript technically sound, and do the data support the conclusions?

The manuscript must describe a technically sound piece of scientific research with data that supports the conclusions. Experiments must have been conducted rigorously, with appropriate controls, replication, and sample sizes. The conclusions must be drawn appropriately based on the data presented.

Reviewer #1: Yes

Reviewer #2: Yes

**********

3. Has the statistical analysis been performed appropriately and rigorously?

Reviewer #1: Yes

Reviewer #2: Yes

**********

4. Have the authors made all data underlying the findings in their manuscript fully available?

The PLOS Data policy requires authors to make all data underlying the findings described in their manuscript fully available without restriction, with rare exception (please refer to the Data Availability Statement in the manuscript PDF file). The data should be provided as part of the manuscript or its supporting information, or deposited to a public repository. For example, in addition to summary statistics, the data points behind means, medians and variance measures should be available. If there are restrictions on publicly sharing data—e.g. participant privacy or use of data from a third party—those must be specified.

Reviewer #1: Yes

Reviewer #2: Yes

**********

5. Is the manuscript presented in an intelligible fashion and written in standard English?

PLOS ONE does not copyedit accepted manuscripts, so the language in submitted articles must be clear, correct, and unambiguous. Any typographical or grammatical errors should be corrected at revision, so please note any specific errors here.

Reviewer #1: Yes

Reviewer #2: Yes

**********

6. Review Comments to the Author

Please use the space provided to explain your answers to the questions above. You may also include additional comments for the author, including concerns about dual publication, research ethics, or publication ethics. (Please upload your review as an attachment if it exceeds 20,000 characters)

Reviewer #1: The manuscript of the article has been significantly improved compared to the previous version, both in the theoretical and in the experimental part. The authors adequately responded to all remarks and comments of the reviewers. The performed corrections removed the observed deficiencies and further improved the quality of work. I believe that the authors have successfully implemented the proposed changes.

Reviewer #2: Thank you for the substantial revisions. You have made considerable efforts to strengthen the theoretical rationale, clarify the study objectives, improve the psychometric evaluation, and revise causal language to better reflect the cross-sectional nature of the study. The validation of the Bangla versions of the SAS-SV and PAUS appears rigorous, and the inclusion of measurement invariance and item response theory analyses adds value to the manuscript.

A few minor issues remain that would benefit from further clarification. These include a brief discussion of the practical significance of the reported indirect effects, consideration of the implications of very high internal consistency estimates, clarification regarding the statement linking sampling diversity to normality assumptions, and further explanation of the consent procedure involving teachers when parents were unavailable. Addressing these points would improve the clarity and interpretation of the findings. These few minor concerns are comments for your consideration in the attached file.

**********

7. PLOS authors have the option to publish the peer review history of their article (what does this mean?). If published, this will include your full peer review and any attached files.

If you choose “no”, your identity will remain anonymous but your review may still be made public.

**Do you want your identity to be public for this peer review?**  For information about this choice, including consent withdrawal, please see our Privacy Policy.

Reviewer #1: No

Reviewer #2: No

**********

Attachment Submitted filename: Review Report_PLOSONE_PONE-D-25-56548_R1.pdf

# Editor report: Acceptance letter

**DOI:** 10.1371/journal.pone.0353865.r004

**Authors:** Gladys Dzansi

PONE-D-25-56548R1

PLOS One

Dear Dr. Shahrier,

I'm pleased to inform you that your manuscript has been deemed suitable for publication in PLOS One. Congratulations! Your manuscript is now being handed over to our production team.

At this stage, our production department will prepare your paper for publication. This includes ensuring the following:

* All references, tables, and figures are properly cited

* All relevant supporting information is included in the manuscript submission,

* There are no issues that prevent the paper from being properly typeset

You will receive further instructions from the production team, including instructions on how to review your proof when it is ready. Please keep in mind that we are working through a large volume of accepted articles, so please give us a few days to review your paper and let you know the next and final steps.

Lastly, if your institution or institutions have a press office, please let them know about your upcoming paper now to help maximize its impact. If they'll be preparing press materials, please inform our press team within the next 48 hours. Your manuscript will remain under strict press embargo until 2 pm Eastern Time on the date of publication. For more information, please contact onepress@plos.org.

You will receive an invoice from PLOS for your publication fee after your manuscript has reached the completed accept phase. If you receive an email requesting payment before acceptance or for any other service, this may be a phishing scheme. Learn how to identify phishing emails and protect your accounts at https://explore.plos.org/phishing.

If we can help with anything else, please email us at customercare@plos.org.

Thank you for submitting your work to PLOS ONE and supporting open access.

Kind regards,

PLOS ONE Editorial Office Staff

on behalf of

Dr. Gladys Dzansi

Academic Editor

PLOS One
